# Thermal response to moderate exercise in dogs of different biotypes and brachycephalic obstructive airway syndrome severity assessed using infrared thermography

**DOI:** 10.3389/fvets.2026.1975908

**Published:** 2026-10-26

**Authors:** Brenda Reyes-Sotelo, Julio Martínez-Burnes, Ismael Hernández-Avalos, Adriana Olmos-Hernández, Patricia Mora-Medina, Dina Villanueva-García, Fabiola Torres-Bernal, Alejandro Casas-Alvarado, Daniel Mota-Rojas

**Affiliations:** 1 Doctoral Program in Biological and Health Sciences, Universidad Autónoma Metropolitana, Mexico City, Mexico; 2 Facultad de Medicina Veterinaria y Zootecnia, Instituto de Ecología Aplicada, Universidad Autónoma de Tamaulipas, Ciudad Victoria, Mexico; 3 Clinical Pharmacology and Veterinary Anesthesia, Biological Sciences Department, Facultad de Estudios Superiores Cuautitlán, Universidad Nacional Autónoma de México, Cuautitlán, Mexico; 4 Department Bioterio and Experimental Surgery, Instituto Nacional de Rehabilitación Luis Guillermo Ibarra Ibarra (INR-LGII), Mexico City, Mexico; 5 Livestock Science Department, Facultad de Estudios Superiores Cuautitlán, Universidad Nacional Autónoma de México, Cuautitlán, Mexico; 6 Division of Neonatology, Hospital Infantil de México Federico Gómez, Mexico City, Mexico; 7 Neurophysiology, Behavior and Animal Welfare Assessment, Department of Agricultural and Animal Production, Universidad Autónoma Metropolitana, Mexico City, Mexico

**Keywords:** BOAS, brachycephalic, dolichocephalic, infrared thermography, mesocephalic

## Abstract

**Introduction:**

The aim of this study was to evaluate dogs of different skull conformations (cephalic biotypes) and Brachycephalic Obstructive Airway Syndrome (BOAS) grades subjected to two standardized moderate exercise tests, in order to determine their effects on infrared thermal responses in facial and appendicular thermal windows.

**Methods:**

A total of 80 dogs were evaluated: dolichocephalic (*n* = 20), mesocephalic (*n* = 20), and brachycephalic (*n* = 40), with the latter classified according to BOAS severity as G_1_ (*n* = 10), G_2_ (*n* = 18), and G_3_ (*n* = 12). Infrared thermal responses were evaluated in facial regions (caruncle, upper and lower eyelids, upper and lower lips, masseter, and nostril region) and appendicular regions (forelimbs, hindlimbs, gracilis, *biceps femoris*, and *gastrocnemius muscles*). Measurements were obtained at four time points: T_1_, baseline; T_2_, after the 6-min walk test; T_3_, after the 1,000-meter walk test; and T_4_, following a 10-min recovery period.

**Results:**

Brachycephalic dogs exhibited significant increases in facial surface temperature, reaching up to 2.6 °C (*p* < 0.0001), and showed slower thermal recovery after 10 min. In G_2_ dogs, muscular thermal windows showed post-exercise temperatures of 35 °C ± 0.5 °C. The 6-min walk test revealed significant differences in distance traveled among cephalic biotypes (*p* = 0.0028). Furthermore, the 1,000-meter walk test revealed more pronounced alterations in superficial microcirculation in brachycephalic dogs across all BOAS grades.

**Discussion:**

The greater facial thermal response and slower recovery observed in brachycephalic dogs suggest potential ventilatory limitations and increased reliance on cutaneous heat dissipation. The two exercise tests provided complementary information, with the 6-min walk test distinguishing differences in initial exercise performance and the 1,000-meter test revealing more pronounced thermal alterations associated with brachycephalic conformation and BOAS severity.

## 1 Introduction

Infrared thermography (IRT) has become established as a non-invasive and highly sensitive tool for evaluating changes associated with exercise, thermal stress (1), and vascular alterations in domestic animals (2). IRT can detect variations in microcirculation, enabling precise analysis of local responses, even in tissues with varying vascular densities (3). Surface temperature is modulated by the interaction among muscular effort, ventilation, peripheral perfusion, and central thermoregulatory mechanisms, all of which depend on integrated neurophysiological pathways, particularly temperature-sensitive ion channels, such as transient receptor potential channel, which are key determinants of thermal perception and reflex vasodilation (4).

In brachycephalic breeds, the thermal assessment of facial structures is particularly relevant due to extreme craniofacial modifications that alter airflow, nostril resistance, and, consequently, ventilatory efficiency (5, 6). Studies such as that of Fernández-Parra et al. (7) have demonstrated, using computational fluid dynamics, increased resistance and intranasal pressures, that compromises evaporative heat dissipation and contribute to superficial thermal accumulation. This effect represents a risk factor during exercise due to increased ventilatory demand and a consequent reduction in air-heat exchange efficiency. During exertion, sensory neurons innervating the airways provide feedback regarding mechanical, chemical and thermal changes within the respiratory tract. These afferent signals are transmitted through vagal sensory pathways to central nervous system circuits that interact with respiratory and autonomic centers, contributing to response regulation (8). Consequently, autonomic adjustments associated with increased respiratory effort may influence peripheral blood flow and cutaneous vascular responses, contributing to changes in surface temperature detected by IRT in facial regions such as the caruncle, eyelids, muzzle, and nostril region. In contrast, appendicular and muscular regions may reflect exercise-induced muscular activity and changes in local blood flow, as reported in dogs subjected to treadmill exercise and 6 min of walking (1, 9).

Standardized exercise tests like the 6-min walk test have proven useful at the clinical level for assessing exercise tolerance in dogs (10–18). They help distinguish between healthy dogs and those with mild to moderate pulmonary conditions, as shown by Swimmer and Rozanski (10), Manens et al. (11), and are also used to monitor distance in dogs with conditions such as congestive heart failure (12), centronuclear myopathy (13), and idiopathic pulmonary fibrosis (14, 15). Conversely, continuous exercise, such as the 1,000 m walk test, has been adopted in brachycephalic breeding programs as a selection criterion. In addition, functional exercise tests, such as the 3-min exercise test described by Liu et al. (19), can be used to assess changes in respiratory function before and after exercise. It allows evaluation of both functional exercise capacity and aerobic performance, as well as clinical signs linked to Brachycephalic Obstructive Airway Syndrome (BOAS), a respiratory disorder related to specific anatomical features of brachycephalic dogs. The severity of BOAS is graded from 0 to 3 based on respiratory symptom intensity (16–18).

Despite advances in infrared thermography and functional assessments, few studies have simultaneously explored the effect of biotype, BOAS severity, and thermal responses. This study aimed to evaluate dogs of different biotypes and BOAS grades subjected to two standardized moderate exercise tests (6 min walk test and 1,000 m walk test), in order to determine their effect on infrared thermal responses in facial thermal windows, including the caruncle, upper and lower eyelids, upper and lower lips, masseter, and nostril region, as well as in appendicular thermal windows, including the forelimbs and hindlimbs, *gracilis muscle, biceps femoralis muscle*, and *gastrocnemius muscle*.

## 2 Materials and methods

### 2.1 Animals and location

A total of 80 purebred and mixed-breed dogs were recruited from 10 veterinary clinics located in the eastern region of Mexico City. The study was reviewed and approved by the Doctoral Committee in Biological and Health Sciences of the Universidad Autónoma Metropolitana, Mexico City (Protocol CBS.205.22). All animals were privately owned companion dogs, and owner authorization was obtained through signed informed consent.

Data were collected from January 2024 to December 2025. Inclusion criteria were dogs 1–6 years of age, with no apparent signs of disease or mobility impairment on general clinical examination and physiological parameters within normal ranges. Breed, age, sex, body weight, and body condition score (BCS) were recorded for each dog. All dogs had up-to-date vaccination and deworming records.

Exclusion criteria: dogs younger than 1 year or older than 6 years; presence of painful or uncomfortable conditions affecting handling; aggressive behavior; and diagnosed infectious diseases or those under treatment at the time of the study. All dogs were classified into three biotypes: B_1_, dolicocephalic (*n* = 20) B_2_, mesocephalic (*n* = 20) and B_3_, brachycephalic (*n* = 40) using the Total Cephalic Index. This latter group was subdivided according to BOAS severity using the pre- and post-exercise functional grading protocol described by Andrews et al. (20). The BOAS functional grading system, which evaluates clinical respiratory responses, was applied as described in the section “Application of BOAS Functional Grading System.” Based on this assessment, dogs were subdivided into G_1_ Grade BOAS 1 (*n* = 10), G_2_ Grade BOAS 2 (*n* = 18), and G_3_ Grade BOAS 3 (*n* = 12). Once TCI and BOAS severity graded had been determined, the dogs underwent two standardized exercise tests, incorporated into the four evaluation time points described in section 2.4.

### 2.2 Determination of the total cephalic index

The Total Cephalic Index (TCI) was calculated using cranial width, measured from the external border of the zygomatic arch to the contralateral border, and cranial length, measured from the external occipital protuberance to the interincisive suture (20). Based on the resulting TCI, dogs were classified as dolichocephalic (TCI < 55%), mesocephalic (TCI 55–80%), or brachycephalic (TCI >80%) (Figure 1).

**Figure 1:**
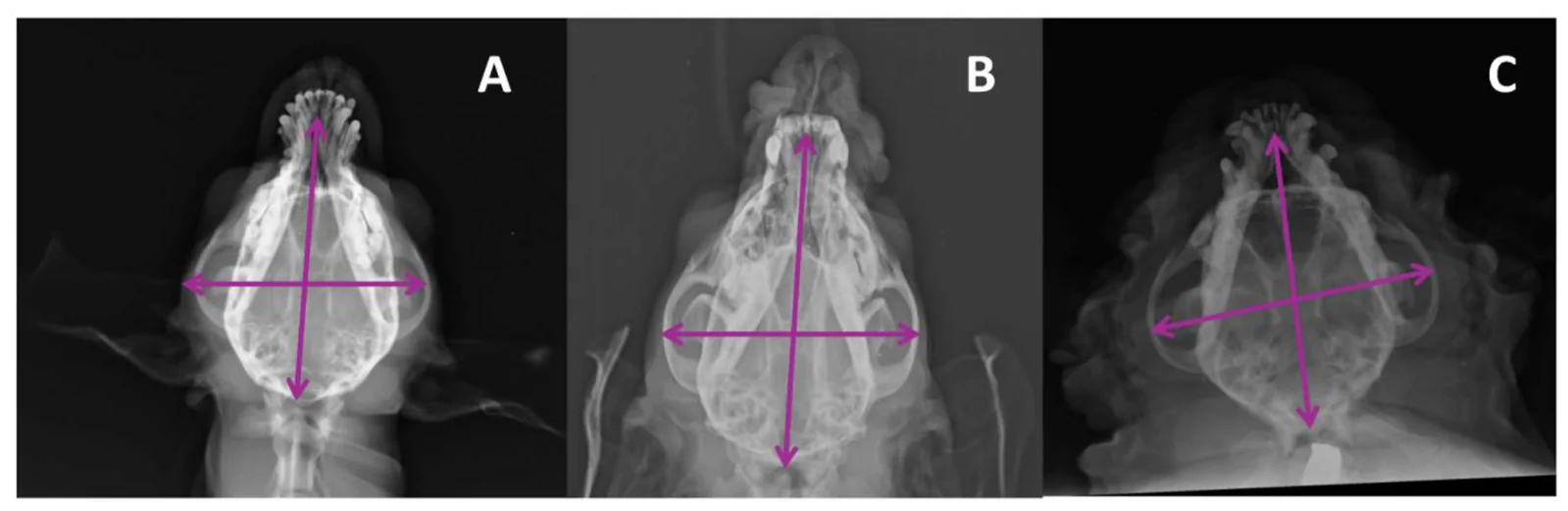
Comparison of skull morphology among canine cephalic biotypes using radiographic images of the head and upper body. The three panels illustrate **(A)** a dolichocephalic dog (TCI < 55%), **(B)** a mesocephalic dog (TCI between 55% and 85%), and **(C)** a brachycephalic dog (TCI > 85%). Bright magenta arrows indicate the vertical and horizontal skull dimensions used to characterize cranial morphology.

Radiographic examinations were performed in all of 80 dogs as part of the research protocol to classify the different cranial conformations based on the TCI. Each dog was evaluated using left lateral and ventrodorsal skull views. Images were acquired with a TXR^®^ X-ray system (Tingle model 325, 125 kV and 300 mA, Vance, AL, USA) integrated with a Fujifilm^®^ digital radiography system (XG5000 plus, Advance Console Version 8.1, Fujifilm Corporation, Tokyo, Japan). The software was used to perform the cranial assessments required to calculate the TCI, using standardized anatomical landmarks and digital tools to improve accuracy and minimize errors.

### 2.3 Application of the BOAS functional grading system

Subdivision of the brachycephalic biotype was established using a standardized exercise test specifically designed for BOAS patients (19). The protocol consisted of a 3-min trot at 6–8 km/h, performed by a trained evaluator without a treadmill.

To minimize external influences, dogs were transported in carriers and assessed 24 h before the standardized exercise testing protocol.

Functional grading was performed according to the criteria described by Liu et al. (19). Dogs classified as Grade 0 exhibited no clinical signs and were considered free of BOAS. Grade 1 dogs displayed mild BOAS, characterized by audible respiratory stertor without evidence of exercise intolerance. Grade 2 corresponded to moderate disease, with clinically relevant signs requiring medical management and potentially corrective surgery. Grade 3 included dogs with severe BOAS with pronounced clinical signs. Based on clinical findings, owners were informed of potential complications and were advised to seek prompt surgical correction.

Brachycephalic dogs were allocated to three study groups: G_1_, control, including Grades 0 and 1; G_2_, Grade 2; and G_3_, Grade 3 BOAS.

### 2.4 Evaluation times and exercise tolerance tests

All 80 dogs distributed among the five groups were subjected to two modified exercise tests adapted from the methodology described by Lilja Maula et al. (14), incorporating the following time points:

Time 1 (T_1_) corresponded to baseline values collected before testing began.

Time 2 (T_2_), recorded immediately after a 6-min walk test conducted along a 40 m covered corridor with natural ventilation. Dogs were led on a leash by a trained handler at a self-selected walking pace without pulling. The test was discontinued if a dog stopped walking, sat down, lay down, or refused to continue, and was considered completed at that point. The test was also terminated if severe dyspnea, cyanosis, syncope, or signs of heat stress were observed. The Total distance covered was measured in meters.

Time 3 (T_3_) was assessed immediately after completion of a 1,000-m walking test conducted under the same conditions as T_2_. Distance and time were recorded in meters and minutes, respectively.

The two exercise assessments were carried out on the same day under uncontrolled environmental conditions. In eastern Mexico City, at an altitude of 2,240 m above sea level, the average temperature between 8 a.m. and 10 a.m. was 16.75 °C, with higher values in April-August (18.6 °C). Mean relative humidity was 61%, with the lowest recorded in March (17%) and the highest in September (98%).

Time 4 (T_4_) represented the recovery period, recorded 10-min after completion of the 1,000-m walking test.

### 2.5 Infrared thermography

Radiometric images were obtained at T_1_ through T_4_ using a FLIR E80 infrared camera (FLIR Systems, Goleta, CA, USA). The device featured an infrared resolution of 320 × 240 pixels, an MSX resolution of 320 x 240 pixels, a thermal sensitivity of < 0.045 °C, and accuracy of ± 2 °C or ± 2 percent at ambient temperatures between 10 °C and 35 °C. The image frequency was 60 Hz and the emissivity standardized at 0.98.

Facial radiometric images were obtained at a distance of 60 to 80 cm, while appendicular radiometric images were captured at 1 m, both at a 90° angle. Thermal images were recorded between 8 a.m. and 10 a.m. in right lateral, left lateral, rostral, and caudal views. The operator used latex gloves during animal handling to optimize precision and prevent thermal contamination.

Four radiometric images were captured at each time point. The first focused on the facial region to obtain seven thermal windows: the lacrimal caruncle, upper and lower eyelids, upper and lower lips, the nostril region, and the masseter. The second and third images corresponded to right and left lateral views to assess appendicular regions, including right and left forelimbs and right and left hindlimbs, with mean values calculated for forelimbs and hindlimbs. The fourth image corresponded to the caudal region, assessing *gracilis muscle, biceps femoralis muscle*, and *gastrocnemius muscle* (Figure 2).

**Figure 2:**
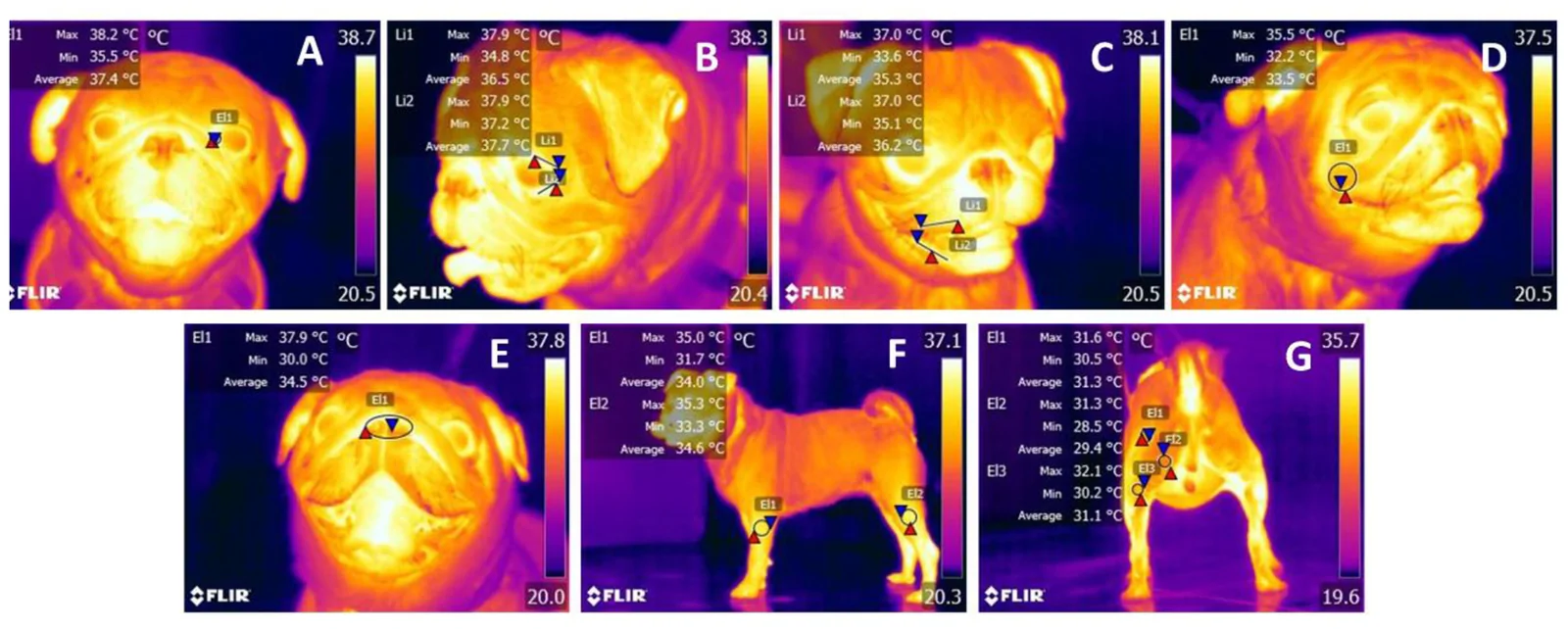
Representation of the twelve thermal windows evaluated in dogs. **(A)** Thermal window of the medial caruncle (${\text{T}}_{\text{caruncle}}^{{}^{\circ}}$) (El1) marked with a circular region of interest (ROI) of 2–5 mm encompasing *supraorbitalis* and *infraorbitalis artheries*. **(B)** Thermal windows of the upper eyelid (${\text{T}}_{\text{Uppereyelid}}^{{}^{\circ}}$); lower eyelid) (${\text{T}}_{\text{lowereyelid}}^{{}^{\circ}}$) (Li1–Li2) drawn as a line betwen 3 and 5 mm that passes through the *angularis oculi* and *supraorbitalis* artheries. **(C)** Thermal windows of the upper lip (${\text{T}}_{\text{Upperlip}}^{{}^{\circ}}$); lower lip) (${\text{T}}_{\text{lowerlip}}^{{}^{\circ}}$) (Li1–Li2) drawn as a line between 3 and 5 mm encompassing *labialis superiori* and deep *temporali* artheries. **(D)** Masseter window (${\text{T}}_{\text{mas}}^{{}^{\circ}}$) (El1) marked with a circular region of interest (ROI) of 5 mm. **(E)** Nostril window (${\text{T}}_{\text{nostril}}^{{}^{\circ}}$) (El1) marked with a circular region 5–7 mm that passes through *lateralis nasi* arthery. **(F)** Forelim region (${\text{T}}_{\text{forelimb}}^{{}^{\circ}}$) (El1); Hindlimb region (${\text{T}}_{\text{hindlmb}}^{{}^{\circ}}$) (El2); marked with a circular region of interest covering 3–5 mm diameter of the limb width encompassing deep antebraquial and saphena artheries. **(G)** Thermal windows of *Biceps femoralis muscle* (${\text{T}}_{\text{femoralis}}^{{}^{\circ}}$) (El1); *Gastrocnemius muscle* (${\text{T}}_{\text{gastrocnemius}}^{{}^{\circ}}$) (El2); *Gracilis muscle* (${\text{T}}_{\text{gracilis}}^{{}^{\circ}}$) (El3) marked with a circular ROI of 3–5 mm that covers *iliaca, femoralis* and *saphena* artheries.

All thermographic images were saved in JPEG format and analyzed using FLIR Tools Software (version 6.4.17317.1002; FLIR Systems, Goleta, CA, USA). For each region of interest, minimum, maximum, and mean temperature values were extracted and recorded.

### 2.6 Statistical analysis

Descriptive statistics were generated using GraphPad Prism software (version 9.5) for all biotypes and BOAS grades across all time points (T_1_-T_4_). Data distribution was examined using a Shapiro-Wilk test to assess the normality assumptions. To evaluate the effects of biotype and time on thermographic measurements, a repeated-measures linear mixed-effects model was fitted. The analysis included the thermal windows corresponding to the ${\text{T}}_{\text{caruncle}}^{{}^{\circ}}$, ${\text{T}}_{\text{uppereyelid}}^{{}^{\circ}}$, ${\text{T}}_{\text{lowereyelid}}^{{}^{\circ}}$, ${\text{T}}_{\text{upperlip}}^{{}^{\circ}}$, ${\text{T}}_{\text{lowerlip}}^{{}^{\circ}}$, ${\text{T}}_{\text{nostril}}^{{}^{\circ}}$, ${\text{T}}_{\text{masseter}}^{{}^{\circ}}$, ${\text{T}}_{\text{forelimbs}}^{{}^{\circ}}$, ${\text{T}}_{\text{hindlimbs}}^{{}^{\circ}}$, T°_*bicepsfemoralis*_, T°_*gastrocnemius*_, and T°_*gracilis*_. When significant effects were detected, Tukey adjusted *post-hoc* pairwise comparisons were performed to account for multiple comparisons. Associations between thermographic variables and physical performance parameters were investigated through Pearson's correlation analysis. Statistical significance was set at *p* < 0.05.

### 2.7 Ethics statement

The experimental protocol received approval from the Academic Committee of the Doctoral Program in Biological and Health Sciences (Approval ID CBS.205.22). All procedures were conducted in accordance with local regulations and institutional requirements. Written informed consent was obtained from all owners for their animals' participation in the study.

## 3 Results

### 3.1 Animals

Animals classified within the dolichocephalic biotype included Dachshund (*n* = 10), mixed breeds (*n* = 4), Chihuahua (*n* = 3), American Bully (*n* = 1), Scottish Terrier (*n* = 1), and Xoloitzcuintli (*n* = 1). Mesocephalic dogs included mixed breeds (*n* = 6), Old English Sheepdog (*n* = 4), Chihuahua (*n* = 3), Labrador Retriever (*n* = 3), Poodle (*n* = 1), American Bull Terrier (*n* = 1), Schnauzer (*n* = 1), and Beagle (*n* = 1).

Brachycephalic dogs included Pug (*n* = 23), Shih Tzu (*n* = 5), English Bulldog (*n* = 4), mixed breeds (*n* = 4), French Bulldog (*n* = 3), and Boston Terrier (*n* = 1). Mean age was 4.28 ± 0.32 years for B_1_, 3.96 ± 0.34 years for B_2_, 4.40 ± 0.50 years for G_1_, 2.81 ± 0.36 years for G_2_, and 3.59 ± 0.55 years for G_3_.

Body weight averaged 7.36 ± 0.76 kg for B_1_, 17.23 ± 2.98 kg for B_2_, 4.40 ± 0.50 kg for G_1_, 8.74 ± 0.80 kg for G_2_, and 10.48 ± 1.71 kg for G_3_.

### 3.2 Physical performance of the three biotypes and BOAS severity groups in brachycephalic dogs during the 6 min and 1,000 m tests

No significant differences were detected at T_2_ between B_1_, B_2_, and B_3_, which achieved mean distances of 462, 451.5, and, 526.22 m, respectively (Kruskal-Wallis, H = 4.54, *p* > 0.05), nor in T_3_ with respect to time (Kruskal-Wallis, H = 3.98, *p* > 0.05). Significant differences among biotypes were observed only in T_3_ distance (Kruskal-Wallis, H = 11.74, *p* = 0.0028).

Within the B_3_ subdivisions, at T_2_, G_1_ covered 552 m, G_2_ 569.83 m, and G_3_ 439.33 m (*p* = 0.0585), without statistically significant differences. Differences were observed only in T_3_ distance, where G_3_ covered 699.1 m compared to G_2_ with 932.77 m (*p* = 0.0389), while G_1_ showed an intermediate value of 876 m without statistically significant difference.

### 3.3 Infrared thermography

Statistically significant differences were observed in minimum (Table 1), maximum (Table 2), and mean surface temperatures of facial thermal windows (Table 3) and appendicular thermal windows in minimum (Table 4), maximum (Table 5), and mean (Table 6) evaluated in 80 dogs across different biotypes and Brachycephalic Obstructive Airway Syndrome grades throughout T_1_ to T_4_.

**Table 1:** Mean and standard error of minimum infrared temperature evaluated in facial thermal windows in different biotypes during moderate exercise program.

Facial thermal windows minimum temperature	B_1_ *n* = 20 mean ±EE	B_2_ *n* = 20 mean ±EE	B_3_			*p*-value
			G_1_ *n* = 10 mean ±EE	G_2_ *n* = 18 mean ±EE	G_3_ *n* = 12 mean ±EE	
${\text{T}}_{\text{lacrimalcaruncle}}^{{}^{\circ}}$						
T_1_	32.74 ± 0.80^a,1^	32.59 ± 0.53^a,c,1^	35.19 ± 0.44^b,1^	34.47 ± 0.30^a,c,1^	33.31 ± 1.04^b,c,1^	***p*** **<** **0.0001**
T_2_	33.25 ± 0.56^a,1^	32.80 ± 0.66^a,1^	35.13 ± 0.47^b,1^	35.37 ± 0.35^b,2^	34.23 ± 0.82^b,1^	***p*** **<** **0.0001**
T_3_	33.39 ± 0.66^a,1^	32.73 ± 0.70^b,1^	35.43 ± 0.31^c,e,1^	35.52 ± 0.29^d,e,2^	34.58 ± 0.87^e,1^	***p*** **<** **0.0001**
T_4_	33.21 ± 0.65^a,1^	32.91 ± 0.73^b,1^	35.15 ± 0.41^c,1^	35.13 ± 0.33^d,e,1,2^	34.45 ± 0.47^e,1^	***p*** **=** **0.0001**
***p*** **-value**	*p* > 0.05	*p* > 0.05	*p* > 0.05	***p*** **<** **0.0001**	*p* > 0.05	
${\text{T}}_{\text{Uppereyelid}}^{{}^{\circ}}$						
T_1_	32.19 ± 0.74^a,1^	31.76 ± 0.62^a,1^	34.26 ± 0.78^b,c,1^	34.14 ± 0.35^c,d,1^	33.49 ± 0.61^d,1^	***p*** **<** **0.0001**
T_2_	32.73 ± 0.69^a,1,2^	31.87 ± 0.81^a,1,2^	34.56 ± 0.59^b,1^	34.62 ± 0.38^a,c,1^	33.13 ± 0.68^c,1^	***p*** **<** **0.0001**
T_3_	33.12 ± 0.71^a,2^	32.64 ± 0.66^a,2^	35.62 ± 0.33^b,1^	35.05 ± 0.35^c,d,2^	34.63 ± 0.50^d,2^	***p*** **=** **0.0004**
T_4_	32.60 ± 0.77^a,1,2^	32.36 ± 0.68^a,1,2^	34.5 ± 0.70^b,c,1^	35.12 ± 0.29^c,d,2^	34.49 ± 0.49^d,1,2^	***p*** **=** **0.0002**
***p*** **-value**	***p*** **=** **0.0008**	***p*** **=** **0.0050**	*p* > 0.05	***p*** **<** **0.0001**	***p*** **=** **0.0031**	
${\text{T}}_{\text{Lowereyelid}}^{{}^{\circ}}$						
T_1_	32.77 ± 0.79^a,1^	31.61 ± 0.81^b,1^	34.64 ± 0.71^c,1^	34.61 ± 0.38^c,1^	34.33 ± 0.41^c,1^	***p*** **<** **0.0001**
T_2_	33.66 ± 0.66^a,1^	33.05 ± 0.74^a,2^	35.14 ± 0.36^a,c,1^	35.63 ± 0.29^b,c, d,2^	33.88 ± 0.56^a,d,1^	***p*** **=** **0.0110**
T_3_	33.70 ± 0.58^a,1^	31.40 ± 1.70^a,b,1,2^	35.76 ± 0.43^b,1^	35.66 ± 0.29^b,2^	34.45 ± 0.87^b,1^	***p*** **<** **0.0001**
T_4_	33.12 ± 0.73^a,1^	32.46 ± 0.80^a,1,2^	35.02 ± 0.68^b,1^	35.23 ± 0.31^b,1,2^	34.08 ± 0.65^b,1^	***p*** **<** **0.0001**
***p*** **-value**	*p* > 0.05	***p*** **=** **0.0021**	*p* > 0.05	***p*** **<** **0.0001**	*p* > 0.05	
${\text{T}}_{\text{Upperlip}}^{{}^{\circ}}$						
T_1_	30.45 ± 0.90^a,1^	30.12 ± 0.88^a,1^	35.57 ± 0.41^b,d,1^	34.46 ± 0.57^c,1^	34.41 ± 0.69^d,1^	***p*** **<** **0.0001**
T_2_	30.60 ± 0.89^a,1^	30.28 ± 1.03^a,1^	35.73 ± 0.39^b,1,2^	35.42 ± 0.31^c,d,1,2^	34.52 ± 0.65^d,1^	***p*** **<** **0.0001**
T_3_	31.68 ± 0.82^a,1^	30.96 ± 0.88^a,1^	36.04 ± 0.48^b,1,2^	35.44 ± 0.42^c,d,2^	34.82 ± 0.66^d,1^	***p*** **<** **0.0001**
T_4_	30.69 ± 0.83^a,1^	30.25 ± 0.92^a,1^	36.34 ± 0.49^b,2^	35.28 ± 0.33^c,d,1,2^	34.95 ± 0.35^d,1^	***p*** **<** **0.0001**
***p*** **-value**	*p* > 0.05	*p* > 0.05	***p*** **=** **0.0467**	***p*** **=** **0.0164**	*p* > 0.05	
${\text{T}}_{\text{Lowerlip}}^{{}^{\circ}}$						
T_1_	30.47 ± 0.79^a,1^	30.64 ± 0.76^a,1^	35.48 ± 0.37^b,d,1^	34.76 ± 0.46^c,d,1^	34.13 ± 0.89^d,1^	***p*** **<** **0.0001**
T_2_	29.93 ± 0.77^a,1,2^	30.39 ± 0.95^a,1,3^	35.89 ± 0.40^b,1,2^	35.59 ± 0.44^c,d,2^	34.10 ± 0.55^d,1^	***p*** **<** **0.0001**
T_3_	32.02 ± 0.65^a,2^	31.38 ± 0.81^a,1,2^	36.48 ± 0.26^b,d,2^	35.27 ± 0.40^c,d,1,2^	35.28 ± 0.60^d,1^	***p*** **<** **0.0001**
T_4_	31.15 ± 0.58^a,1^	31.88 ± 0.70^a,2^	36.50 ± 0.27^b,2^	35.08 ± 0.45^c,d,1,2^	34.93 ± 0.48^d,1^	***p*** **<** **0.0001**
***p*** **-value**	***p*** **<** **0.0001**	***p*** **<** **0.0001**	***p*** **=** **0.0467**	***p*** **=** **0.0467**	*p* > 0.05	
${\text{T}}_{\text{Masseter}}^{{}^{\circ}}$						
T_1_	30.94 ± 0.81^a,1^	29.10 ± 0.87^b,1^	32.25 ± 0.65^c,1^	32.8 ± 0.36^a,c,1^	31.52 ± 1.59^a,c,1^	***p*** **<** **0.0001**
T_2_	30.96 ± 0.62^a,1^	28.99 ± 0.84^b,1,3^	32.66 ± 0.47^c,1^	33.51 ± 0.29^c,d,2^	31.73 ± 1.50^a,c,d,1^	***p*** **<** **0.0001**
T_3_	31.57 ± 0.80^a,1^	29.29 ± 0.76^b,1,3^	32.94 ± 0.55^c,1,2^	33.78 ± 0.49^c,d,2^	31.24 ± 1.46^a,c,d,1^	***p*** **<** **0.0001**
T_4_	31.07 ± 0.77^a,1^	29.83 ± 0.70^b,1,2^	33.73 ± 0.50^c,2^	33.49 ± 0.35^d,2^	30.67 ± 1.21^a,d,1^	***p*** **<** **0.0001**
***p*** **-value**	*p* > 0.05	***p*** **=** **0.0120**	***p*** **<** **0.0001**	***p*** **=** **0.0001**	*p* > 0.05	
${\text{T}}_{\text{nostril}}^{{}^{\circ}}$						
T_1_	21.60 ± 0.60^a,1^	24.80 ± 0.74^a,c,1^	24.00 ± 0.90^a,c1^	27.00 ± 0.96 ^b,c,1^	26.7 ± 1.24^a,c,1^	***p*** **=** **0.0009**
T_2_	22.60 ± 0.92^a,1^	27.90 ± 1.01^a,c,1,2^	28.80 ± 0.84^a,b, c,2^	30.10 ± 1.04 ^b,c,1^	29.30 ± 0.82^b,c,1^	***p*** **=** **0.0132**
T_3_	22.60 ± 0.892^a,1^	27.80 ± 0.87^b,2^	29.00 ± 0.96^a,b,1,2^	29.60 ± 0.76^b,1^	30.40 ± 0.61^a,b,1^	***p*** **=** **0.0017**
T_4_	21.70 ± 0.84^a,1^	26.30 ± 1.13^a,c,1,2^	28.30 ± 0.82^a,b, c,1,2^	28.90 ± 0.85^b,c,1^	29.20 ± 0.76^a,b, c,1^	***p*** **=** **0.0059**
***p*** **-value**	*p* > 0.05	***p*** **=** **0.0206**	***p*** **=** **0.0024**	*p* > 0.05	*p* > 0.05	

*n*, number of animals; EE, standard error; B1, dolichocephalic; B2, mesocephalic; B3, brachycephalic; G-1, BOAS Grade 1; G-2, BOAS Grade 2; G-3, BOAS Grade 3.

^a,b,c,d,e^different letters indicate significant differences *p* < 0.05 between columns.

^1,2,3^different number indicate significant differences *p* < 0.05 between lines.

Bold values indicate statistically significant differences (*p* < 0.05).

**Table 2:** Mean and standard error of maximum infrared temperature evaluated in facial thermal windows in different biotypes during moderate exercise program.

Facial thermal windows maximum temperature	B_1_ *n* = 20 mean ±EE	B_2_ *n* = 20 mean ±EE	B_3_			*p*-value
			G_1_ *n* = 10 mean ±EE	G_2_ *n* = 18 mean ±EE	G_3_ *n* = 12 mean ±EE	
${\text{T}}_{\text{lacrimalcaruncle}}^{{}^{\circ}}$						
T_1_	35.81 ± 0.35^a,1^	35.91 ± 0.30^a,1^	37.03 ± 0.36^b,c,1^	36.67 ± 0.24^c,1^	36.80 ± 0.25^d,b,1^	***p*** **<** **0.0001**
T_2_	36.25 ± 0.27^a,1,2^	35.93 ± 0.40^a,1^	37.24 ± 0.39^b,c,d,1^	37.39 ± 0.18^c,2^	37.15 ± 0.29^c,d,1,2^	***p*** **<** **0.0001**
T_3_	36.54 ± 0.30^a,2^	36.13 ± 0.47^a,b,c,d,1^	37.62 ± 0.26^b,d,1^	37.24 ± 0.24^c,2^	37.58 ± 0.22^d,2^	***p*** **<** **0.0001**
T_4_	35.90 ± 0.38^a,1^	35.79 ± 0.57^a,b,d,1^	37.36 ± 0.36^b,c,d,1^	37.08 ± 0.29^c,1,2^	37.23 ± 0.21^d,2^	***p*** **<** **0.0001**
***p*** **-value**	***p*** **=** **0.0004**	*p* > 0.05	*p* > 0.05	***p*** **<** **0.0001**	***p*** **<** **0.0001**	
${\text{T}}_{\text{Uppereyelid}}^{{}^{\circ}}$						
T_1_	35.03 ± 0.39^a,1^	34.63 ± 0.46^b,1^	36.30 ± 0.56^c,e,1^	35.61 ± 0.30^d,1^	36.39 ± 0.36^e,1^	***p*** **<** **0.0001**
T_2_	35.19 ± 0.37^a,1^	34.99 ± 0.61^a,c,1^	36.74 ± 0.34^b,1^	36.36 ± 0.23^c,d,2^	36.20 ± 0.42^d,1^	***p*** **<** **0.0001**
T_3_	35.51 ± 0.34^a,2^	35.31 ± 0.51^a,b, c,d,1^	37.02 ± 0.26^b,1^	36.41 ± 0.26^c,2^	36.75 ± 0.28^d,1^	***p*** **<** **0.0001**
T_4_	34.81 ± 0.54^a,1,2^	35.08 ± 0.57^a,1^	36.64 ± 0.43^b,d,1^	36.36 ± 0.34^c,2^	36.47 ± 0.30^d,1^	***p*** **<** **0.0001**
***p*** **-value**	***p*** **=** **0.0102**	*p* > 0.05	*p* > 0.05	***p*** **<** **0.0001**	***p*** **<** **0.0001**	
${\text{T}}_{\text{Lowereyelid}}^{{}^{\circ}}$						
T_1_	34.86 ± 0.41^a,1^	34.77 ± 0.56^a,b, c,1^	36.69 ± 0.35^b,d,1^	36.17 ± 0.27^c,d,1^	36.28 ± 0.37^d,1^	***p*** **<** **0.0001**
T_2_	35.70 ± 0.32^a,2^	35.41 ± 0.53^a,c,2^	36.90 ± 0.22^b,d,1,2^	36.74 ± 0.18^c,d,2^	36.42 ± 0.39^d,1^	***p*** **<** **0.0001**
T_3_	35.55 ± 0.41^a,2^	35.11 ± 0.71^a,b, c,1,2^	37.25 ± 0.25^b,1,3^	36.26 ± 0.34^a,1,2,3^	36.97 ± 0.23^c,1^	***p*** **=** **0.0001**
T_4_	35.12 ± 0.44^a,1^	35.19 ± 0.59^a,1,2^	36.47 ± 0.64^b,c,d,1,2,3^	36.56 ± 0.29^c,d, 1, 2,4^	36.39 ± 0.36^d,1^	***p*** **<** **0.0001**
***p*** **-value**	***p*** **<** **0.0001**	***p*** **=** **0.0040**	***p*** **=** **0.0084**	***p*** **=** **0.0027**	*p* > 0.05	
${\text{T}}_{\text{Upperlip}}^{{}^{\circ}}$						
T_1_	32.89 ± 0.69^a,1^	33.16 ± 0.58^a,1^	37.01 ± 0.40^b,d,1^	35.90 ± 0.45^c,d,1^	35.38 ± 0.86^d,1^	***p*** **<** **0.0001**
T_2_	33.89 ± 0.49^a,2^	33.72 ± 0.69^a,1^	37.41 ± 0.28^b,1,2^	36.91 ± 0.31^c,d,2^	36.45 ± 0.48^d,1^	***p*** **<** **0.0001**
T_3_	34.41 ± 0.52^a,3^	33.49 ± 0.74^a,1^	37.63 ± 0.30^b,2^	36.88 ± 0.34^c,2^	36.80 ± 0.28^d,1^	***p*** **<** **0.0001**
T_4_	33.31 ± 0.56^a,1,2^	34.16 ± 0.40^a,1^	37.13 ± 0.50^c,e,1,2^	36.58 ± 0.34^d,e,2^	36.52 ± 0.34^e,1^	***p*** **<** **0.0001**
***p*** **-value**	***p*** **<** **0.0001**	*p* > 0.05	***p*** **=** **0.0138**	***p*** **=** **0.0066**	*p* > 0.05	
${\text{T}}_{\text{Lowerlip}}^{{}^{\circ}}$						
T_1_	32.36 ± 0.69^a,1^	34.07 ± 0.39^b,1^	36.75 ± 0.34^c,e,1^	35.89 ± 0.45^d,e,1^	35.66 ± 0.80^e,1^	***p*** **<** **0.0001**
T_2_	33.50 ± 0.51^a,2^	33.92 ± 0.62^a,1,2^	37.17 ± 0.18^b,1,2^	36.53 ± 0.36^c,d,2^	35.93 ± 0.39^d,1,2^	***p*** **<** **0.0001**
T_3_	34.29 ± 0.53^a,3^	34.29 ± 0.60^a,1,2^	37.36 ± 0.21^b,d,1,3^	36.56 ± 0.33^c,2^	36.88 ± 0.24^d,1,3^	***p*** **<** **0.0001**
T_4_	33.97 ± 0.49^a,2,3^	34.80 ± 0.41^b,2^	37.33 ± 0.27^c,2,3^	36.42 ± 0.33^d,1,2^	36.55 ± 0.17^e,1,2^	***p*** **<** **0.0001**
***p*** **-value**	***p*** **<** **0.0001**	***p*** **<** **0.0001**	***p*** **=** **0.0200**	***p*** **=** **0.0358**	***p*** **=** **0.0288**	
${\text{T}}_{\text{Masseter}}^{{}^{\circ}}$						
T_1_	33.66 ± 0.71^a,1^	31.48 ± 0.78^b,1^	35.08 ± 0.60^c,1^	35.03 ± 0.27^a,c,1^	33.78 ± 1.53^a,c,1^	***p*** **<** **0.0001**
T_2_	33.61 ± 0.51^a,1^	32.26 ± 0.84^a,d,1,2^	35.51 ± 0.41^b,c,e,1^	35.78 ± 0.35^c,e,2^	34.46 ± 1.26^a,e,1^	***p*** **<** **0.0001**
T_3_	33.81 ± 0.62^a,1^	32.71 ± 0.71^b,2^	35.75 ± 0.55^c,d,e,1^	35.96 ± 0.32^d,e,2^	34.71 ± 1.18^e,1^	***p*** **=** **0.0002**
T_4_	33.35 ± 0.62^a,1^	32.69 ± 0.68^b,2^	35.62 ± 0.50^c,d,e,1^	35.76 ± 0.29^d,e,2^	34.65 ± 1.19^e,1^	***p*** **<** **0.0001**
***p*** **-value**	*p* > 0.05	***p*** **=** **0.0001**	*p* > 0.05	***p*** **<** **0.0001**	*p* > 0.05	
${\text{T}}_{\text{nostril}}^{{}^{\circ}}$						
T_1_	31.00 ± 0.60^a,1^	33.1 ± 0.65^a,c1^	33.70 ± 0.70^a,c1^	35.50 ± 0.70^b,c,1^	34.10 ± 1.22^a,c,1^	***p*** **=** **0.0106**
T_2_	32.20 ± 0.83^a,1^	35.30 ± 0.57^a,c,1^	36.40 ± 0.43^a,c1^	36.60 ± 0.61^b,c,1^	31.2 ± 0.70^a,c,1^	***p*** **=** **0.0104**
T_3_	31.20 ± 0.70^a,1^	35.00 ± 0.66^a,c,1^	36.60 ± 0.29^b,c,1^	36.30 ± 0.33^b,c,1^	37.00 ± 0.29^a,c1^	***p*** **=** **0.0017**
T_4_	30.70 ± 0.76^a,1^	33.60 ± 0.79^a,c.1^	36.00 ± 0.51^a,c1^	35.90 ± 0.35^b,c,1^	36.2 ± 0.30^a,c,1^	***p*** **=** **0.0120**
***p*** **-value**	*p* > 0.05	*p* > 0.05	*p* > 0.05	*p* > 0.05	*p* > 0.05	

*n*, number of animals; EE, standard error; B1, dolichocephalic; B2, mesocephalic; B3, brachycephalic; G-1, BOAS Grade 1; G-2, BOAS Grade 2; G-3, BOAS Grade 3.

^a,b,c,d,e^different letters indicate significant differences *p* < 0.05 between columns.

^1,2,3,4^different number indicate significant differences *p* < 0.05 between lines.

Bold values indicate statistically significant differences (*p* < 0.05).

**Table 3:** Mean and standard error of average infrared temperature evaluated in facial thermal windows in different biotypes during moderate exercise program.

Facial thermal windows average temperature	B1 *n* = 20 mean ±EE	B2 *n* = 20 media ±EE	B3			*p*-value
			G1 *n* = 10 media ±EE	G2 *n* = 18 media ±EE	G3 *n* = 12 media ±EE	
${\text{T}}_{\text{lacrimalcaruncle}}^{{}^{\circ}}$						
T1	34.43 ± 0.59^a,1^	34.50 ± 0.38^a,1^	36.39 ± 0.36^b,1^	35.91 ± 0.24^c,d,1^	35.53 ± 0.50^d,1^	***p*** **<** **0.0001**
T2	34.80 ± 0.42^a,1^	34.54 ± 0.57^a,1^	36.38 ± 0.50^b,c,d,1^	36.66 ± 0.18^c,d,2^	36.11 ± 0.45^d,1,2^	***p*** **<** **0.0001**
T3	35.15 ± 0.46^a,1^	34.78 ± 0.55^a,1^	36.90 ± 0.27^b,d,1^	36.58 ± 0.26^c,d,2^	36.48 ± 0.37^d,2^	***p*** **=** **0.0001**
T4	34.77 ± 0.46^a,1^	34.54 ± 0.62^a,1^	36.45 ± 0.37^b,c,d,1^	36.37 ± 0.29^c,d,2^	36.28 ± 0.23^d,1,2^	***p*** **<** **0.0001**
***p*** **-value**	*p* > 0.05	*p* > 0.05	*p* > 0.05	***p*** **<** **0.0001**	***p*** **=** **0.0039**	
${\text{T}}_{\text{Uppereyelid}}^{{}^{\circ}}$						
T1	33.79 ± 0.55^a,1^	33.38 ± 0.49^a,1^	35.33 ± 0.67^b,c,d,1^	34.99 ± 0.31^c,d,1^	35.05 ± 0.48^d,1^	***p*** **<** **0.0001**
T2	34.05 ± 0.48^a,1,2^	33.55 ± 0.66^a,1,2^	35.70 ± 0.36^b,1^	35.64 ± 0.26^c,d,2^	34.78 ± 0.51^d,2^	***p*** **=** **0.0004**
T3	34.29 ± 0.54^a,2^	34.20 ± 0.55^a,2,3^	36.28 ± 0.20^b,1^	35.90 ± 0.29^c,d,2,3^	35.74 ± 0.35^d,1,3^	***p*** **<** **0.0001**
T4	33.73 ± 0.65^a,1,2^	34.03 ± 0.60^a,2,3^	35.63 ± 0.66^b,c,d,1^	35.86 ± 0.32^c,d,2,3^	35.53 ± 0.38^d,1,2,3^	***p*** **<** **0.0001**
***p*** **-value**	***p*** **=** **0.0003**	***p*** **=** **0.0014**	*p* > 0.05	***p*** **<** **0.0001**	***p*** **=** **0.0086**	
${\text{T}}_{\text{Lowereyelid}}^{{}^{\circ}}$						
T1	33.95 ± 0.56^a,1^	33.45 ± 0.69^a,1^	35.76 ± 0.45^b,d,1^	35.58 ± 0.31^c,d,1^	35.57 ± 0.35^d,1^	***p*** **<** **0.0001**
T2	34.79 ± 0.39^a,2^	34.43 ± 0.59^a,2^	36.27 ± 0.22^b,d,1,2^	36.33 ± 0.20^c,d,2^	35.40 ± 0.47^d,1,2^	***p*** **<** **0.0001**
T3	34.63 ± 0.48^a,2^	34.33 ± 0.74^a,b,c,d,2^	36.59 ± 0.24^b,d,1,3^	36.27 ± 0.26^c,d,2^	36.00 ± 0.37^d,1,3^	***p*** **=** **0.0002**
T4	34.31 ± 0.52^a,1,2^	34.18 ± 0.66^a,1,2^	35.73 ± 0.67^b,c,d,1,2,3^	36.04 ± 0.29^c,d,1,2^	35.69 ± 0.34^d,1,2,3^	***p*** **<** **0.0001**
***p*** **-value**	***p*** **=** **0.0087**	***p*** **=** **0.0028**	***p*** **=** **0.0018**	***p*** **=** **0.0001**	***p*** **=** **0.0340**	
${\text{T}}_{\text{Upperlip}}^{{}^{\circ}}$						
T1	31.77 ± 0.82^a,1^	31.69 ± 0.77^a,1^	36.55 ± 0.40^b,1^	35.28 ± 0.50^c,1^	35.14 ± 0.62^d,1^	***p*** **<** **0.0001**
T2	32.40 ± 0.69^a,1,2^	31.97 ± 0.87^a,1^	36.72 ± 0.31^b,1^	36.36 ± 0.30^c,d,2^	35.62 ± 0.55^d,1^	***p*** **<** **0.0001**
T3	33.10 ± 0.65^a,2^	32.24 ± 0.80^a,1^	36.99 ± 0.30^b,1^	36.32 ± 0.41^c,d,2^	35.89 ± 0.48^d,1^	***p*** **<** **0.0001**
T4	32.09 ± 0.70^a,1,2^	32.20 ± 0.66^a,1^	36.8 ± 0.50^b,d,1^	35.96 ± 0.31^c,d,1,2^	35.84 ± 0.28^d,1^	***p*** **<** **0.0001**
***p*** **-value**	***p*** **=** **0.0172**	*p* > 0.05	*p* > 0.05	***p*** **=** **0.0049**	*p* > 0.05	
${\text{T}}_{\text{Lowerlip}}^{{}^{\circ}}$						
T1	31.55 ± 0.71^a,1^	32.8 ± 0.44^b,1^	36.22 ± 0.33^c,e,1^	35.24 ± 0.54^d,e,1^	34.98 ± 0.85^e,1^	***p*** **<** **0.0001**
T2	31.79 ± 0.60^a,1,3^	32.35 ± 0.71^a,1,3^	36.65 ± 0.23^b,1,2^	36.11 ± 0.39^c,d,2^	35.45 ± 0.50^d,1^	***p*** **<** **0.0001**
T3	33.34 ± 0.57^a,2^	33.1 ± 0.64^a,1,4^	37.07 ± 0.23^b,d,2,3^	35.97 ± 0.35^c,1,2^	36.13 ± 0.36^d,1^	***p*** **<** **0.0001**
T4	32.79 ± 0.46^a,1,4^	33.53 ± 0.55^a,2,4^	37.00 ± 0.21^b,1,3^	35.87 ± 0.36^c,1,2^	35.89 ± 0.21^d,1^	***p*** **<** **0.0001**
***p*** **-value**	***p*** **<** **0.0001**	***p*** **=** **0.0010**	***p*** **<** **0.0001**	***p*** **=** **0.0277**	*p* > 0.05	
${\text{T}}_{\text{Masseter}}^{{}^{\circ}}$						
T1	32.19 ± 0.78^a,1^	30.25 ± 0.80^b,1^	33.62 ± 0.62^c,1^	33.98 ± 0.29^a,c,1^	32.63 ± 1.58^a,c,1^	***p*** **<** **0.0001**
T2	32.24 ± 0.57^a,1^	30.74 ± 0.77^b,1,2^	34.20 ± 0.38^c,d,1,2^	34.67 ± 0.30^d,2^	32.90 ± 1.31^a,c,d,1^	***p*** **<** **0.0001**
T3	32.66 ± 0.66^a,1^	31.30 ± 0.73^b,2^	34.48 ± 0.55^c,d,2^	34.92 ± 0.39^d,2^	33.08 ± 1.34^a,c,d,1^	***p*** **<** **0.0001**
T4	32.19 ± 0.74^a,1^	31.42 ± 0.66^a,2^	34.94 ± 0.51^b,c,d,2^	34.49 ± 0.31^c,d,2^	33.76 ± 1.16^d,1^	***p*** **<** **0.0001**
***p*** **-value**	*p* > 0.05	***p*** **<** **0.0001**	***p*** **=** **0.0043**	***p*** **<** **0.0001**	*p* > 0.05	
${\text{T}}_{\text{perinasal}}^{{}^{\circ}}$						
T1	24.70 ± 0.68^a,1^	28.00 ± 0.82^a,c,1^	28,10 ± 0.88^a,c,1^	31.00 ± 0.93^b,c,1^	30.30 ± 1.32^a,c,1^	***p*** **=** **0.0001**
T2	26.20 ± 0.97^a,1^	31.40 ± 0.96^a,b,c,1,3^	32.70 ± 0.56^a,b,c,2,3^	33.40 ± 0.92^b,1^	33.20 ± 0.55^c,1^	***p*** **=** **0.0082**
T3	24.90 ± 0.81^a,1^	31.40 ± 0.93^b,c,2,3^	32.70 ± 0.68^b,c,1,3^	33.40 ± 0.43^b,c1^	34.20 ± 0.39^e,1^	***p*** **<** **0.0001**
T4	24.90 ± 0.85^a,1^	29.60 ± 1.10^a,b,1,3^	32.20 ± 0.89^a,b,1,3^	32.80 ± 0.70^b,1^	33.40 ± 0.61^b,1^	***p*** **<** **0.0001**
***p*** **-value**	*p* > 0.05	***p*** **=** **0.0129**	***p*** **=** **0.0085**	*p* > 0.05	*p* > 0.05	

*n*, number of animals; EE, standard error; B1, dolichocephalic; B2, mesocephalic; B3, brachycephalic; G-1, BOAS Grade 1; G-2, BOAS Grade 2; G-3, BOAS Grade 3.

^a,b,c,d,e^different letters indicate significant differences *p* < 0.05 between columns.

^1,2,3,4^different number indicate significant differences *p* < 0.05 between lines.

Bold values indicate statistically significant differences (*p* < 0.05).

**Table 4:** Mean and standard error of minimum infrared temperature evaluated in appendicular thermal windows in different biotypes during moderate exercise program.

Appendicular thermal windows minimum temperature	B_1_ *n* = 20 mean ±EE	B_2_ *n* = 20 mean ±EE	B_3_			*p*-value
			G_1_ *n* = 10 mean ±EE	G_2_ *n* = 18 mean ±EE	G_3_ *n* = 12 mean ±EE	
${\text{T}}_{\text{Forelimbs}}^{{}^{\circ}}$						
T_1_	31.23 ± 0.66^a,1^	30.00 ± 0.72^b,1^	31.41 ± 0.45^c,d,1^	32.73 ± 0.45^d,1^	28.33 ± 1.02^a,b,1^	***p*** **<** **0.0001**
T_2_	31.24 ± 0.85^a,1^	30.26 ± 0.68^b,1^	31.81 ± 0.56^c,d,1^	33.57 ± 0.46^d,2^	29.06 ± 0.69^a,1^	***p*** **<** **0.0001**
T_3_	32.45 ± 0.64^a,2^	31.20 ± 0.71^b,2^	31.68 ± 0.80^c,d,1^	33.14 ± 0.49^d,1^	29.38 ± 1.12^a,b,d,1^	***p*** **<** **0.0001**
T_4_	30.70 ± 0.68^a,1^	30.81 ± 0.62^a,2^	31.54 ± 0.78^b,c,1^	33.56 ± 0.42^c,2^	28.73 ± 0.90^a,1^	***p*** **<** **0.0001**
***p*** **-value**	***p*** **<** **0.0001**	***p*** **<** **0.0001**	*p* > 0.05	***p*** **=** **0.0037**	*p* > 0.05	
${\text{T}}_{\text{Hidllimbs}}^{{}^{\circ}}$						
T_1_	32.41 ± 0.72^a,1^	30.05 ± 0.70^b,1^	31.93 ± 0.75^c,1^	32.51 ± 0.44^a,c,1^	27.88 ± 0.89^d,b,1^	***p*** **<** **0.0001**
T_2_	31.72 ± 0.85^a,1^	30.58 ± 0.77^b,1,2^	32.79 ± 0.61^c,d,1^	34.09 ± 0.32^d,2^	29.16 ± 1.00^a,b,1^	***p*** **<** **0.0001**
T_3_	32.11 ± 0.77^a,1^	31.14 ± 0.73^b,1^	32.68 ± 0.65^c,d,1^	34.05 ± 0.45^d,2,3^	29.62 ± 1.07^a,b,1^	***p*** **<** **0.0001**
T_4_	31.74 ± 0.71^a,1^	30.86 ± 0.64^b,1^	32.46 ± 0.60^c,d,1^	33.45 ± 0.32^d,3^	29.25 ± 1.15^a,b,d,1^	***p*** **<** **0.0001**
***p*** **-value**	*p* > 0.05	***p*** **=** **0.0015**	*p* > 0.05	***p*** **<** **0.0001**	*p* > 0.05	
T°*_*gracilis*_*						
T_1_	30.79 ± 0.82^a,1^	29.67 ± 0.59^a,c,1^	30.33 ± 0.78^b,1^	31.44 ± 0.66^a,d,1^	28.45 ± 0.99^a,d,1^	***p*** **<** **0.0001**
T_2_	30.65 ± 0.90^a,1^	30.14 ± 0.67^a,d, 1,2,3^	31.54 ± 0.78^b,c,2^	32.92 ± 0.51^c,2,3^	29.28 ± 0.92^d,1,2^	***p*** **<** **0.0001**
T_3_	31.34 ± 0.73^a,1^	30.46 ± 0.65^a,2^	31.42 ± 0.83^b,c,1,2^	33.69 ± 0.54^c,2,4^	30.21 ± 1.04^a,c,1,3^	***p*** **<** **0.0001**
T_4_	31.47 ± 0.74^a,1^	29.58 ± 0.59^b,1,2,3^	32.01 ± 0.74^c,2^	32.43 ± 0.61^d,2,3^	28.70 ± 1.09^a,b, d,1,2^	
***p*** **-value**	*p* > 0.05	***p*** **=** **0.0007**	***p*** **=** **0.0003**	***p*** **<** **0.0001**	***p*** **=** **0.0117**	
T°_*biicepsfemoralis*_						
T_1_	30.21 ± 0.79^a,1^	29.24 ± 0.56^a,1^	30.09 ± 0.44^b,c,1^	30.20 ± 0.40^c,1^	28.06 ± 0.70^a,c,1^	***p*** **<** **0.0001**
T_2_	30.45 ± 0.80^a,1,2^	29.63 ± 0.64^a,1,2^	30.86 ± 0.53^b,c,2^	32.10 ± 0.46^c,2^	28.36 ± 0.62^a,c,1^	***p*** **<** **0.0001**
T_3_	31.17 ± 0.79^a,2^	29.69 ± 0.63^a,2,3^	31.19 ± 0.66^b,c,1,2^	32.14 ± 0.53^c,2^	29.28 ± 0.93^a,b, c,1^	***p*** **<** **0.0001**
T_4_	31.04 ± 0.74^a,2^	28.82 ± 0.68^a,1, 2,4^	30.48 ± 0.74^c,1,2^	30.89 ± 0.57^a,c,d,1^	28.68 ± 0.84^a,b,e,1^	***p*** **<** **0.0001**
***p*** **-value**	***p*** **<** **0.0001**	***p*** **=** **0.0050**	*p* > 0.05	***p*** **<** **0.0001**	*p* > 0.05	
T°*_*gastrocnemio*_*						
T_1_	29.92 ± 0.76^a,1^	29.30 ± 0.68^a,1^	29.84 ± 0.55^b,c,1^	30.63 ± 0.52^c,1^	28.97 ± 0.99^a,c,1^	***p*** **<** **0.0001**
T_2_	30.51 ± 0.84^a,1,2^	29.76 ± 0.64^a,1,2^	31.13 ± 0.67^b,c,2^	31.94 ± 0.52^c,2^	28.75 ± 0.96^a,c,1^	***p*** **<** **0.0001**
T_3_	31.02 ± 0.73^a,2^	29.84 ± 0.63^a,2,3^	30.69 ± 0.85^b,c,1,2^	32.12 ± 0.57^c,2^	30.51 ± 1.22^a,b, c,1^	***p*** **<** **0.0001**
T_4_	30.86 ± 0.77^a,2^	28.99 ± 0.63^a,1, 2,4^	31.24 ± 0.76^c,1,2^	31.12 ± 0.55^a,c,d,1^	29.31 ± 1.18^a,b,e,1^	***p*** **<** **0.0001**
***p*** **-value**	***p*** **=** **0.0006**	***p*** **=** **0.0031**	***p*** **=** **0.0155**	***p*** **<** **0.0001**	*p* > 0.05	

*n*, number of animals; EE, standard error; B1, dolichocephalic; B2, mesocephalic; B3, brachycephalic; G-1, BOAS Grade 1; G-2, BOAS Grade 2; G-3, BOAS Grade 3.

^a,b,c,d,e^different letters indicate significant differences *p* < 0.05 between columns.

^1,2,3,4^different number indicate significant differences *p* < 0.05 between lines.

Bold values indicate statistically significant differences (*p* < 0.05).

**Table 5:** Mean and standard error of maximum infrared temperature evaluated in facial thermal windows in different biotypes during moderate exercise program.

Appendicular thermal windows maximum temperature	B_1_ *n* = 20 mean ±EE	B_2_ *n* = 20 mean ±EE	B_3_			*p*-value
			G_1_ *n* = 10 mean ±EE	G_2_ *n* = 18 mean ±EE	G_3_ *n* = 12 mean ±EE	
${\text{T}}_{\text{Forelimbs}}^{{}^{\circ}}$						
T_1_	34.31 ± 0.49^a,1^	32.28 ± 0.64^b,d,1^	33.48 ± 0.49^a,c,1^	34.72 ± 0.38^c,1^	30.48 ± 0.84^d,1^	***p*** **<** **0.0001**
T_2_	34.20 ± 0.57^a,1^	32.91 ± 0.64^b,1,2^	33.40 ± 0.53^a,c,d,1^	35.43 ± 0.35^c,2^	31.98 ± 0.57^a,b, e,2^	***p*** **<** **0.0001**
T_3_	35.19 ± 0.27^a,1,2^	33.35 ± 0.61^b,2^	34.65 ± 0.60^a,c,2^	35.22 ± 0.42^a,e,1,2^	31.91 ± 0.77^a,b, d,1,2^	***p*** **<** **0.0001**
T_4_	33.75 ± 0.60^a,1,3^	33.10 ± 0.53^b,1,2^	34.38 ± 0.36^a,c,1,2^	35.21 ± 0.36^c,e,1,2^	31.22 ± 0.72^a,b, d,1,2^	***p*** **<** **0.0001**
***p*** **-value**	***p*** **=** **0.0382**	***p*** **=** **0.0220**	***p*** **=** **0.0341**	***p*** **=** **0.0188**	***p*** **=** **0.0487**	
${\text{T}}_{\text{Hindlimbs}}^{{}^{\circ}}$						
T_1_	34.69 ± 0.51^a,1^	32.30 ± 0.71^b,c,1^	33.78 ± 0.65^a,1^	34.01 ± 0.40^a,1^	30.59 ± 0.69^c,1^	***p*** **<** **0.0001**
T_2_	33.85 ± 0.61^a,1,3^	33.69 ± 0.55^a,2^	34.39 ± 0.49^b,c,1^	35.43 ± 0.32^c,2^	32.10 ± 0.85^a,c,1^	***p*** **<** **0.0001**
T_3_	34.52 ± 0.58^a,1,4^	33.70 ± 0.53^a,d,2,3^	34.13 ± 0.40^a,d,1^	35.89 ± 0.32^b,d, c,2^	32.26 ± 0.96^a,c,1^	***p*** **=** **0.0085**
T_4_	33.99 ± 0.48^a,2,4^	33.22 ± 0.56^b,2,4^	34.51 ± 0.37^c,d,1^	35.30 ± 0.28^d,2^	31.54 ± 0.97^a,b,d,1^	***p*** **=** **0.0007**
***p*** **-value**	***p*** **=** **0.0007**	***p*** **<** **0.0001**	*p* > 0.05	***p*** **<** **0.0001**	*p* > 0.05	
T°*_*gracilis*_*						
T_1_	33.27 ± 0.69^a,1^	32.43 ± 0.60^b,1^	33.82 ± 0.46^a,b, c,d,1^	34.68 ± 0.51^a,c,1^	31.13 ± 0.96^a,b, c,1^	***p*** **=** **0.0008**
T_2_	33.80 ± 0.72^a,1^	33.08 ± 0.61^a,1^	33.46 ± 0.58^b,c,1^	35.29 ± 0.39^c,1,2^	31.80 ± 0.81^a,b,1^	***p*** **<** **0.0001**
T_3_	34.14 ± 0.56^a,1^	33.39 ± 0.50^b,1,2^	34.51 ± 0.50^c,d,2^	35.94 ± 0.46^d,2,3^	32.23 ± 0.92^a,b,1,2^	***p*** **<** **0.0001**
T_4_	34.12 ± 0.53^a,1^	32.82 ± 0.50^b,1,3^	34.82 ± 0.71^c,d,1,2^	34.94 ± 0.37^d,1, 2,4^	31.00 ± 0.97^a,b,1,3^	***p*** **<** **0.0001**
***p*** **-value**	*p* > 0.05	***p*** **=** **0.0057**	***p*** **=** **0.0214**	***p*** **<** **0.0001**	***p*** **=** **0.0166**	
T°*_*bicepsfemoralis*_*						
T_1_	32.68 ± 0.57^a,1^	31.52 ± 0.56^ab,1^	31.73 ± 0.63 ^a,c,1^	32.14 ± 0.42^a,c,d,1^	29.71 ± 0.70^a,b,d,1^	***p*** **=** **0.0001**
T_2_	32.85 ± 0.68^a,1,2^	32.26 ± 0.61^b,2^	32.65 ± 0.50 ^c,d,1^	33.99 ± 0.44^d,2^	29.95 ± 0.49^a,b,1,2^	***p*** **<** **0.0001**
T_3_	33.19 ± 0.61^a,2^	32.44 ± 0.57^a,2^	32.86 ± 0.64^b,c,1^	34.16 ± 0.49^c,,2^	31.93 ± 0.82 ^a,c,2,3^	***p*** **<** **0.0001**
T_4_	33.16 ± 0.56^a,1,2^	32.08 ± 0.60^b,2^	32.66 ± 0.52 ^c,1^	49.82 ± 17.2^a,b1,2^	31.64 ± 0.87^a,b,2,3^	***p*** **<** **0.0001**
***p*** **-value**	***p*** **=** **0.0043**	***p*** **=** **0.0005**	*p* > 0.05	***p*** **<** **0.0001**	***p*** **=** **0.0006**	
T°*_*gastrocnemio*_*						
T_1_	32.88 ± 0.69^a,1^	32.11 ± 0.70^a,c,1^	32.44 ± 0.60^b,1^	33.59 ± 0.38^a,b,d,1^	31.29 ± 1.04^a,c,e,1^	***p*** **<** **0.0001**
T_2_	33.12 ± 0.80^a,1^	32.87 ± 0.60^a,c,2^	33.22 ± 0.89^b,d,1^	34.75 ± 0.31^a,d,2^	30.79 ± 0.80^a.c.1.2^	***p*** **=** **0.0003**
T_3_	33.39 ± 0.66^a,1^	32.87 ± 0.63^a,b,2^	33.10 ± 0.88^b,c,d,1^	34.97 ± 0.48^c,d,2^	33.47 ± 0.81^d,2^	***p*** **=** **0.0004**
T_4_	33.64 ± 0.68^a,1^	32.58 ± 0.56^a,c,1,2^	33.87 ± 0.84^b,d,1^	33.86 ± 0.45^a,d,2^	32.48 ± 1.06^a,c, d,1,2^	***p*** **=** **0.0003**
***p*** **-value**	*p* > 0.05	***p*** **=** **0.0020**	*p* > 0.05	***p*** **<** **0.0001**	***p*** **=** **0.0024**	

*n*, number of animals; EE, standard error; B1, dolichocephalic; B2, mesocephalic; B3, brachycephalic; G-1, BOAS Grade 1; G-2, BOAS Grade 2; G-3, BOAS Grade 3.

^a,b,c,d,e^different letters indicate significant differences *p* < 0.05 between columns.

^1,2,3,4^different number indicate significant differences *p* < 0.05 between lines.

Bold values indicate statistically significant differences (*p* < 0.05).

**Table 6:** Mean and standard error of average infrared temperature evaluated in facial thermal windows in different biotypes during moderate exercise program.

Appendicular thermal windows average temperature	B1 *n* = 20 mean ±EE	B2 *n* = 20 mean ±EE	B3			*p*-value
			G1 *n* = 10 mean ±EE	G2 *n* = 18 mean ±EE	G3 *n* = 12 mean ±EE	
${\text{T}}_{\text{Forelimbs}}^{{}^{\circ}}$						
T1	32.81 ± 0.59^a,1^	30.49 ± 0.81^b,1^	32.57 ± 0.42^a,c,1^	33.86 ± 0.39^c,1^	29.44 ± 0.93^a,b,1^	***p*** **<** **0.0001**
T2	32.87 ± 0.70^a,1^	31.61 ± 0.72^b,2^	32.85 ± 0.61^c,d,1^	34.89 ± 0.36^d,2^	30.60 ± 0.67^a,b,1^	***p*** **<** **0.0001**
T3	34.00 ± 0.43^a,2^	32.25 ± 0.70^b,3^	33.40 ± 0.57^a,c,1^	34.32 ± 0.42^c,3^	30.61 ± 0.95^a,d,1^	***p*** **=** **0.0002**
T4	34.00 ± 0.43^a,2^	31.37 ± 0.64^b,2,4^	33.44 ± 0.4^c,d,1^	34.52 ± 0.37^d,1,2,3^	29.93 ± 0.81^e,1^	***p*** **<** **0.0001**
***p*** **-value**	***p*** **=** **0.0022**	***p*** **<** **0.0001**	*p* > 0.05	***p*** **=** **0.0004**	*p* > 0.05	
${\text{T}}_{\text{Hindlimbs}}^{{}^{\circ}}$						
T1	33.60 ± 0.61^a,1^	31.24 ± 0.70^b,d,1^	32.92 ± 0.66^c,1^	33.34 ± 0.41^a,c,1^	29.27 ± 0.81^d,1^	***p*** **<** **0.0001**
T2	32.70 ± 0.77^a,1,2^	32.21 ± 0.66^a,2^	33.99 ± 0.64^b,c,2^	34.93 ± 0.29^c,2^	30.88 ± 0.96^a,1^	***p*** **<** **0.0001**
T3	33.50 ± 0.67^a,1,3^	32.63 ± 0.62^a,2^	33.48 ± 0.52^b,c,1,2^	35.12 ± 0.37^c,2^	31.07 ± 1.06^a,1^	***p*** **=** **0.0020**
T4	32.84 ± 0.60^a,2,3^	32.10 ± 0.61^b,2^	33.68 ± 0.42^c,d,1,2^	34.67 ± 0.30^d,2^	30.43 ± 1.10^a,b,d,1^	***p*** **<** **0.0001**
***p*** **-value**	***p*** **=** **0.0015**	***p*** **<** **0.0001**	***p*** **=** **0.0001**	***p*** **<** **0.0001**	*p* > 0.05	
T°*_*gracilis*_*						
T1	32.05 ± 0.76^a,1^	30.87 ± 0.60^b,1^	32.53 ± 0.54^c,d,1^	33.51 ± 0.52^d,1^	29.79 ± 1.02^a,b,d,1^	***p*** **<** **0.0001**
T2	32.81 ± 0.82^a,2^	31.51 ± 0.62^b,2^	32.62 ± 0.57^c,d,1^	34.41 ± 0.40^d,2^	30.48 ± 0.87^a,b,1^	***p*** **=** **0.0001**
T3	32.79 ± 0.67^a,2^	31.80 ± 0.54^b,2,3^	33.07 ± 0.69^c,d,1^	35.05 ± 0.49^d,3^	31.26 ± 1.01^a,b,1,2^	***p*** **<** **0.0001**
T4	32.81 ± 0.65^a,1,2^	31.12 ± 0.52^b,1, 2,4^	33.61 ± 0.76^c,d,1^	33.87 ± 0.45^d,1,4^	29.73 ± 1.08^a,b,1,3^	***p*** **<** **0.0001**
***p*** **-value**	***p*** **=** **0.0140**	***p*** **<** **0.0001**	*p* > 0.05	***p*** **<** **0.0001**	***p*** **=** **0.0133**	
T°*_*bicepsfemoralis*_*						
T1	31.56 ± 0.67^a,1^	30.40 ± 0.54^b,1^	30.85 ± 0.50^c,1^	31.21 ± 0.41^a,c,d,1^	28.94 ± 0.69^a,b,d,1^	***p*** **=** **0.0043**
T2	31.84 ± 0.74^a,1,2^	31.02 ± 0.61^b,2^	31.77 ± 0.44^c,d,1,2^	33.00 ± 0.41^d,2^	29.17 ± 0.54^a,b,1^	***p*** **<** **0.0001**
T3	32.11 ± 0.70^a,2^	30.98 ± 0.56^a,1,2^	32.07 ± 0.64^b,c,2^	33.27 ± 0.52^c,2,3^	30.58 ± 0.81^a,c, 2,4^	***p*** **<** **0.0001**
T4	32.10 ± 0.66^a,1,2^	30.84 ± 0.46^b,1,2^	31.78 ± 0.71^c,1,2^	31.91 ± 0.55^a,c, d,1,3^	30.15 ± 0.82^a,b, d, 3,4^	***p*** **<** **0.0001**
***p*** **-value**	***p*** **=** **0.0050**	***p*** **=** **0.0416**	***p*** **=** **0.0464**	***p*** **<** **0.0001**	***p*** **=** **0.0107**	
${\text{T}}_{\text{gastrocnemio}}^{{}^{\circ}}$						
T1	31.33 ± 0.73^a,1^	30.55 ± 0.69^a,c,1^	31.26 ± 0.55^b,d,1^	32.26 ± 0.42^a,d,1^	30.30 ± 1.04^a,b, c,d,1^	***p*** **<** **0.0001**
T2	31.89 ± 0.83^a,1,2^	31.23 ± 0.58^a,2^	32.34 ± 0.70^b,c,1^	33.34 ± 0.42^c,2^	29.94 ± 0.88^a,c,1^	***p*** **=** **0.0004**
T3	32.78 ± 0.74^a,2^	31.36 ± 0.65^b,2^	32.07 ± 0.87^c,d,1^	33.66 ± 0.55^d,2,3^	31.92 ± 1.01^a,b, c, d,2,3^	***p*** **=** **0.0010**
T4	32.27 ± 0.73^a,2^	30.75 ± 0.56^b,1,2^	32.56 ± 0.79^c,1^	32.53 ± 0.47^a,c, d, 1, 2,4^	31.00 ± 1.14^a,b, d,1,3^	***p*** **<** **0.0001**
***p*** **-value**	***p*** **=** **0.0005**	***p*** **=** **0.0001**	*p* > 0.05	***p*** **<** **0.0001**	***p*** **=** **0.0005**	

*n*, number of animals; EE, standard error; B1, dolichocephalic; B2, mesocephalic; B3, brachycephalic; G-1, BOAS Grade 1; G-2, BOAS Grade 2; G-3, BOAS Grade 3.

^a,b,c,d,e^different letters indicate significant differences *p* < 0.05 between columns.

^1,2,3,4^different number indicate significant differences *p* < 0.05 between lines.

Bold values indicate statistically significant differences (*p* < 0.05).

The B_3_ group showed significantly higher mean temperatures across most facial and appendicular thermal windows than B_1_ and B_2_ (*p* < 0.0001). Additionally, G_2_ and G_3_ exhibited greater post-exercise increases at T_2_ and T_3_.

The correlation tables are provided in the Supplementary material.

#### 3.3.1 Facial thermal windows in different biotypes subjected to moderate exercise

Mean temperature, as well as minimum (Tables 1 and 3) and maximum (Table 4) values for ${\text{T}}_{\text{caruncle}}^{{}^{\circ}}$ showed significant differences among all biotypes at all time points (*p* < 0.0001; Figure 3). At T_1_, B_1_ and B_2_ presented values between 34.4 °C and 34.5 °C, whereas brachycephalic dogs showed higher values, particularly G_1_ (36.39 °C ± 0.36 °C).

**Figure 3:**
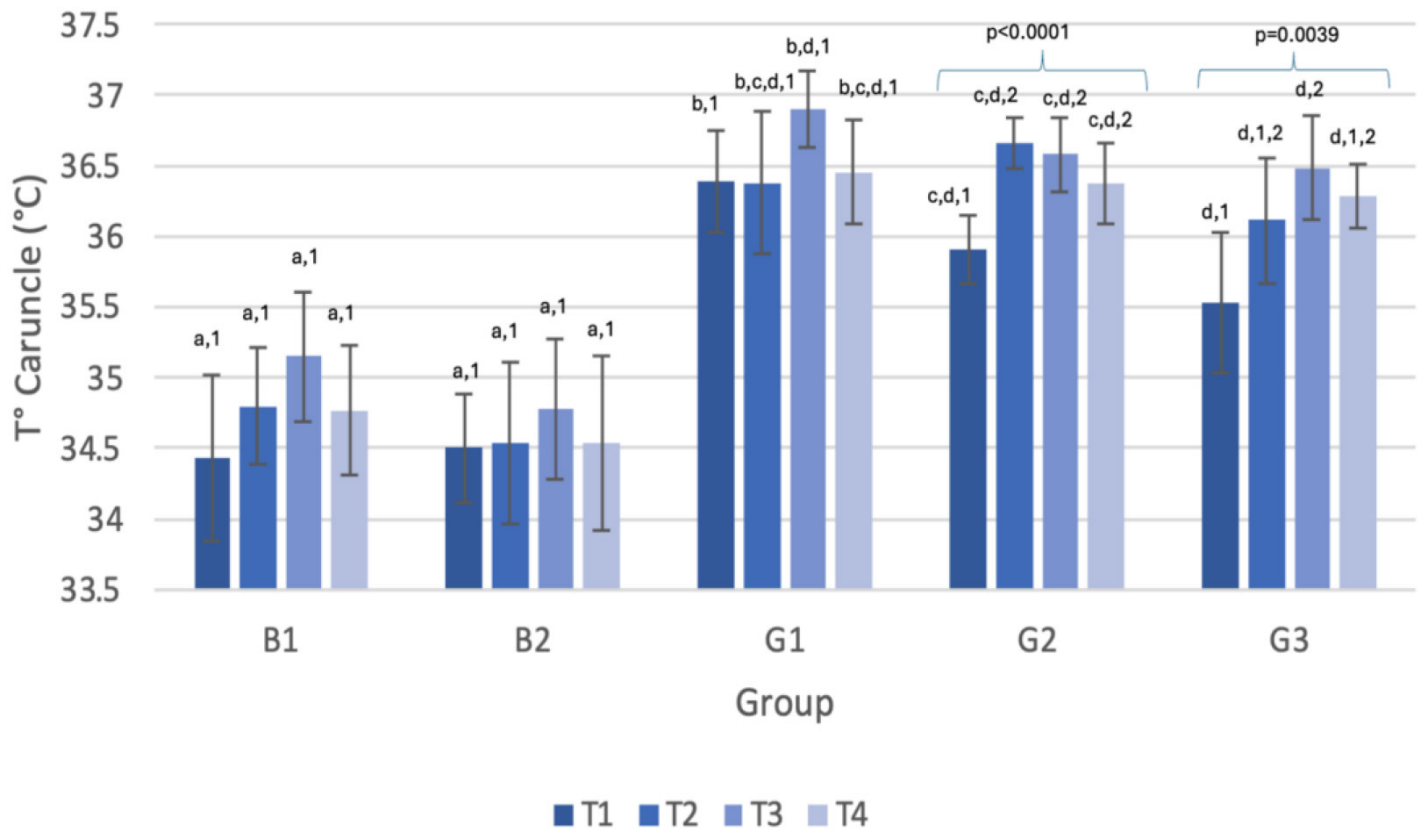
Mean and standard error of caruncle temperature in dogs of different biotypes and BOAS severity in four evaluation times. B_1_, dolichocephalic; B_2_, mesocephalic; B_3_, brachycephalic; G_1_, BOAS Grade 1; G_2_, BOAS Grade 2; G_3_, BOAS Grade 3. ^a,b, c, d^different letters indicate significant differences *p* < 0.05 between groups. ^1, 2^different number indicate significant differences *p* < 0.05 between times.

At T_2_ and T_3_, B_1_ and B_3_ showed increases between 0.3 °C and 0.6 °C. G_1_ recorded the highest temperature at T_3_ (36.90 °C ± 0.27 °C), with statistically significant differences (*p* = 0.0001) compared to the other biotypes.

For ${\text{T}}_{\text{uppereyelid}}^{{}^{\circ}}$ and ${\text{T}}_{\text{lowereyelid}}^{{}^{\circ}}$, B_3_ consistently exhibited higher temperatures, with statistically significant differences compared to B_1_ and B2 (*p* < 0.001) at all evaluation times. At T_1_, values for G_1_, G_2_, and G_3_ ranged between 35.0 °C and 35.7 °C, compared to 33.3 °C to 33.8 °C in B_1_ and B_2_. Following T_2_ and T_3_, significant increases were observed in B_3_ across all grades, reaching values between 36.2 °C and 36.9 °C.

For ${\text{T}}_{\text{upperlip}}^{{}^{\circ}}$ and ${\text{T}}_{\text{lowerlip}}^{{}^{\circ}}$, B_1_ and B_2_ showed baseline temperatures between 31.6 °C and 32.8 °C, whereas B_3_ presented mean values between 35.1 °C and 36.5 °C. At T_2_ and T_3_, groups G_1_ to G_3_ exhibited increases of approximately +1 °C, with elevated temperatures persisting at T_4_ in G_3_ (36.13 °C ± 0.36 °C).

For ${\text{T}}_{\text{masseter}}^{{}^{\circ}}$, statistically significant differences were observed at all time points (*p* < 0.0001). B_1_ and B_2_ ranged between 29 and 32 °C, whereas G_1_ to G_3_ reached mean and maximum values between 33 °C and 35 °C.

Finally, ${\text{T}}_{\text{nostril}}^{{}^{\circ}}$ showed the greatest thermal variation. At T_1_, B_2_ and B_3_ (G_1_-G_3_) presented the highest values (33.10 °C ± 0.65 °C, 33.70 °C ± 0.70 °C, 35.50 °C ± 0.70 °C and 34.10 °C ±1.22 °C, respectively; *p* = 0.0106). At T_2_ and T_3_, temperature increased in B_3_ subdivisions, with G_3_ showing the highest increase at T_3_ (37.00 °C ± 0.29 °C), compared to B_1_ (31.20 °C ± 0.70 °C) and B_2_ (35.00 °C ± 0.66 °C) (*p* < 0.0017; Figure 4).

**Figure 4:**
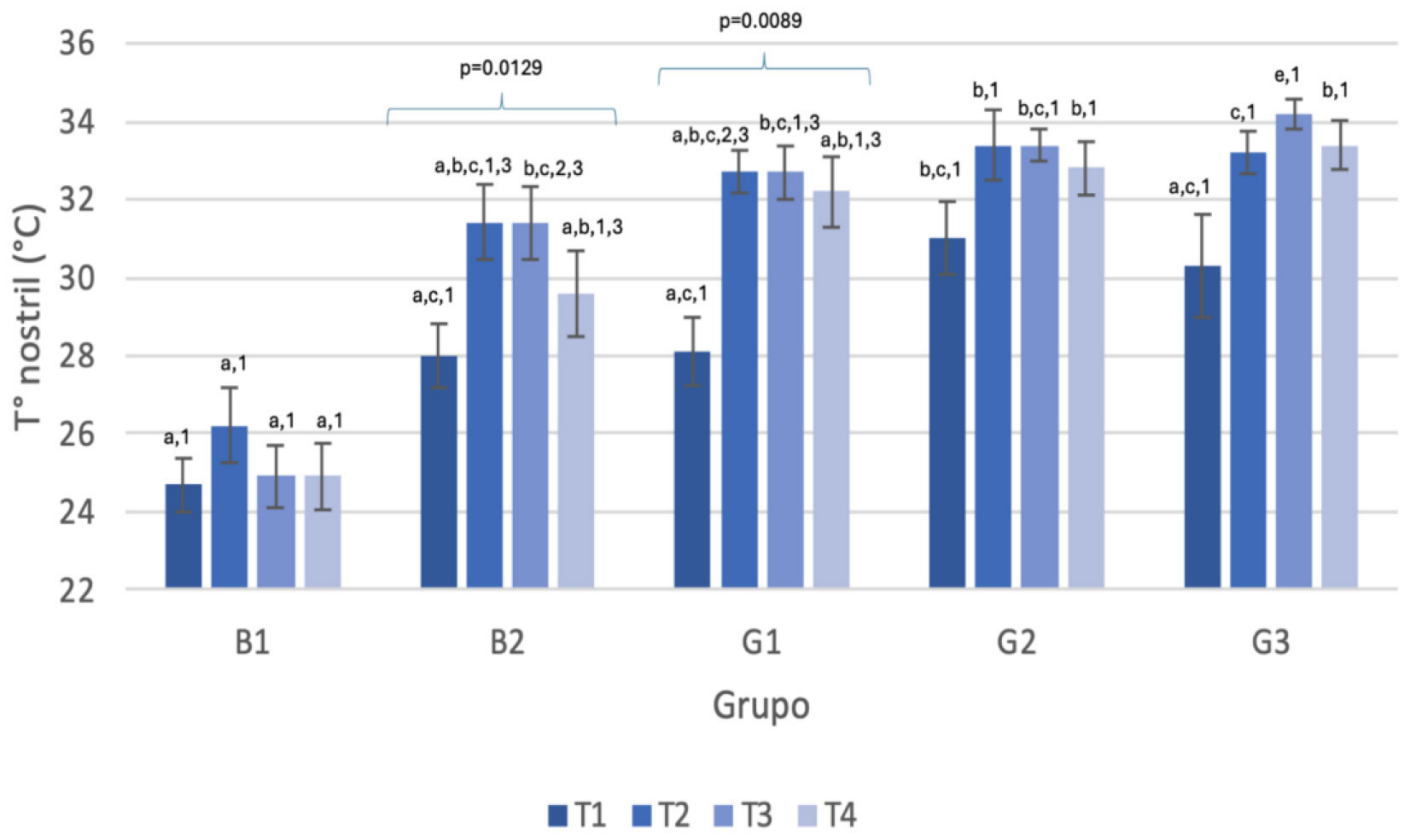
Mean and standard error of nostril temperature in dogs of different biotypes and BOAS severity in four evaluation times. B_1_, dolichocephalic; B_2_, mesocephalic; B_3_, brachycephalic; G_1_, BOAS Grade 1; G_2_, BOAS Grade 2; G_3_, BOAS Grade 3. ^a,b, c, e^different letters indicate significant differences *p* < 0.05 between groups. ^1, 2, 3^different number indicate significant differences *p* < 0.05 between times.

#### 3.3.2 Appendicular thermal windows in different biotypes subjected to moderate exercise

Minimum, maximum, and mean temperatures for appendicular thermal windows are shown in Tables 4–6. ${\text{T}}_{\text{forelimbs}}^{{}^{\circ}}$ revealed that G_3_ had the lowest baseline value at T_1_ (29.44 °C ± 0.93 °C) compared with other groups, with no statistically significant differences over time. However, significant increases were observed at T_2_ and T_3_ in G_2_ (34.89 °C ± 0.36 °C and 34.32 °C ± 0.42 °C, respectively; *p* = 0.0004), which persisted at T_4_ (34.52 °C ± 0.37 °C; Figure 5).

**Figure 5:**
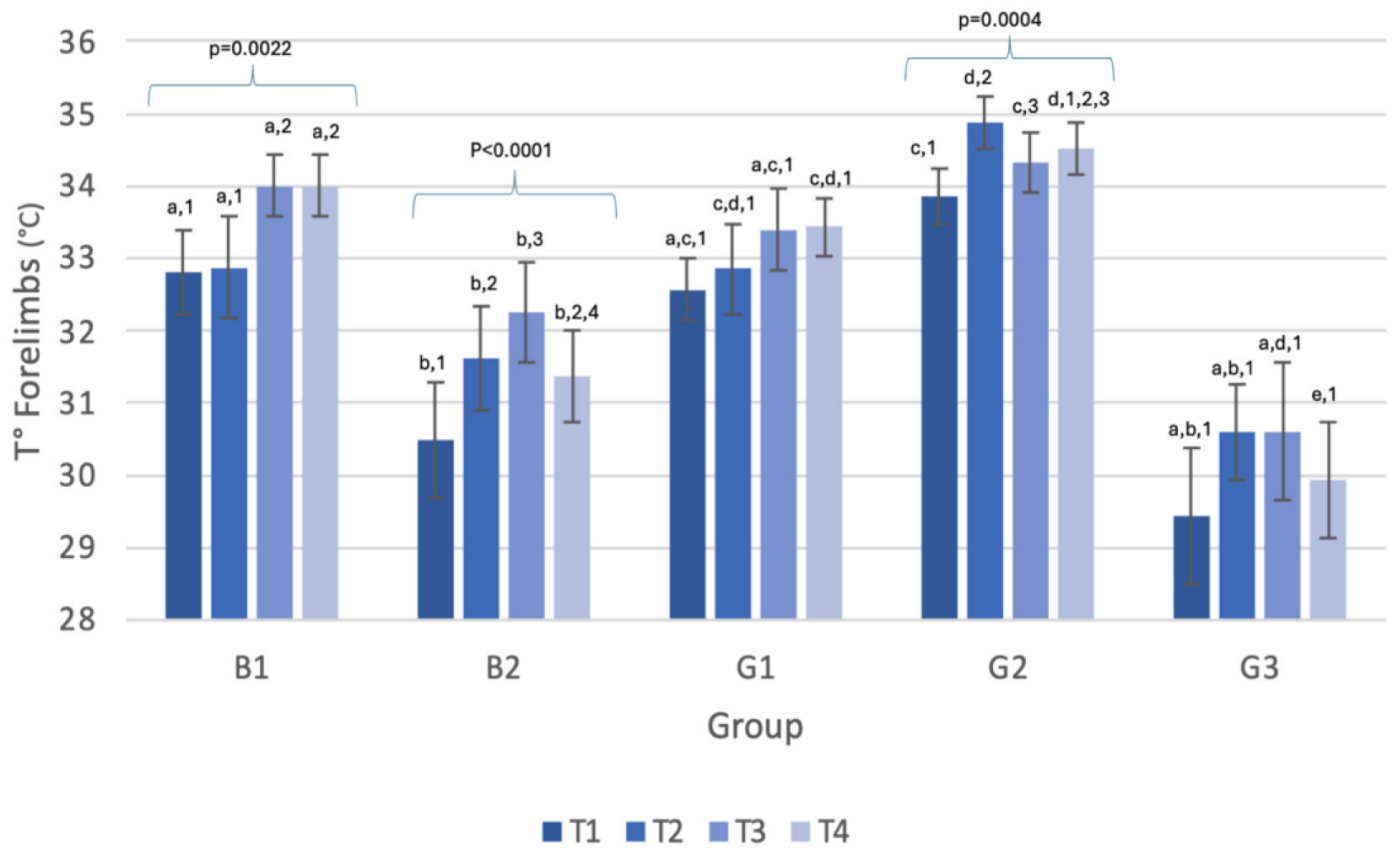
Mean and standard error of forelimbs temperature in dogs of different biotypes and BOAS severity in four evaluation times. B_1_, dolichocephalic; B_2_, mesocephalic; B_3_, brachycephalic; G_1_, BOAS Grade 1; G_2_, BOAS Grade 2; G_3_, BOAS Grade 3. ^a,b, c, d, e^different letters indicate significant differences *p* < 0.05 between groups. ^1, 2, 3, 4^different number indicate significant differences *p* < 0.05 between times.

A similar pattern was observed for ${\text{T}}_{\text{hindlimbs}}^{{}^{\circ}}$, where G_3_ again showed the lowest baseline value (29.27 °C ± 0.81 °C). In contrast, G_1_ and G_2_ exhibited increases between 34 °C and 35 °C following T_2_ and T_3_, whereas B_1_ and B_2_ remained between 30 °C and 32 °C across all time points (Figure 6).

**Figure 6:**
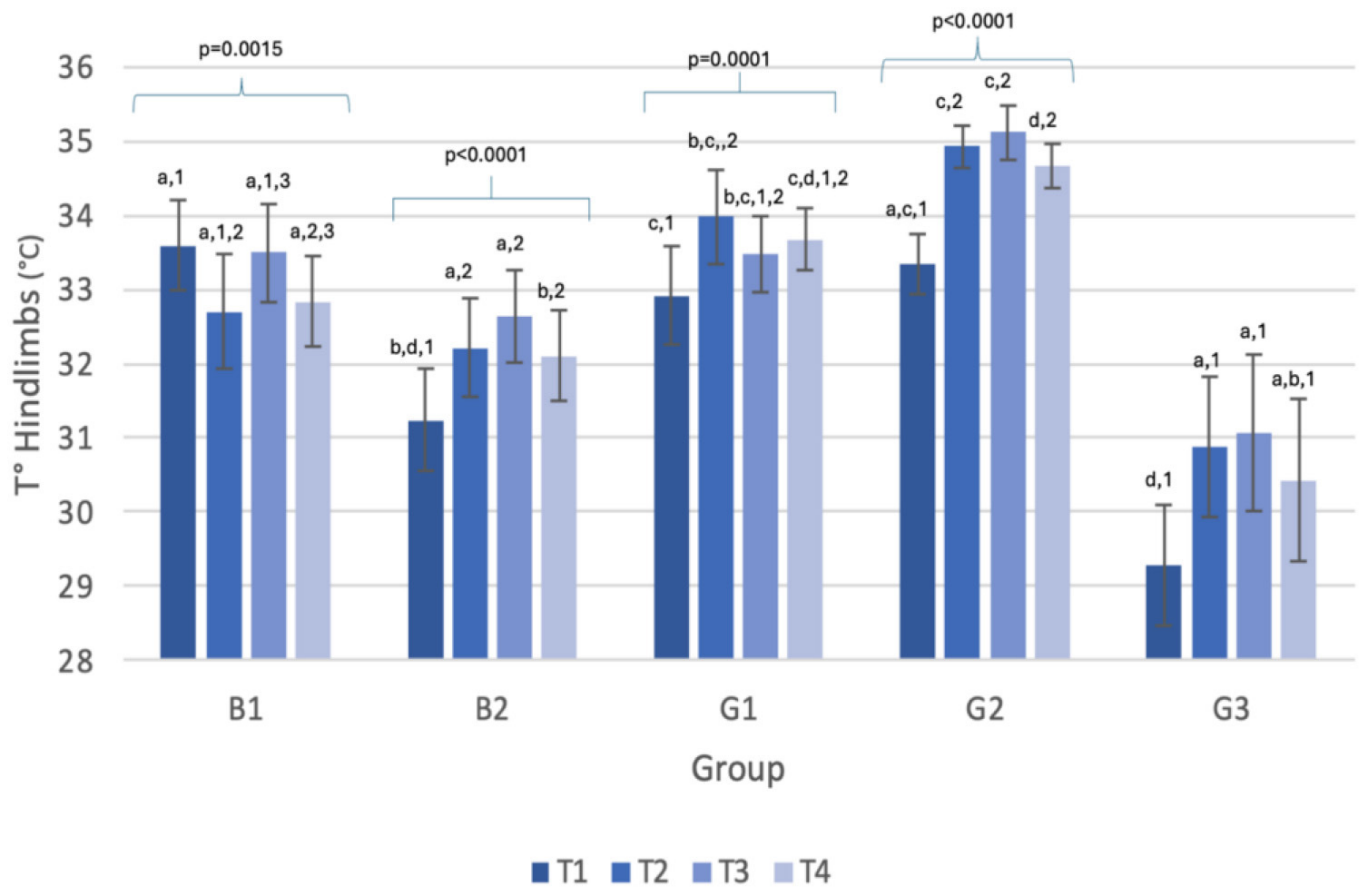
Mean and standard error of hindlimbs temperature in dogs of different biotypes and BOAS severity in four evaluation times. B_1_, dolichocephalic; B_2_, mesocephalic; B_3_, brachycephalic; G_1_, BOAS Grade 1; G_2_, BOAS Grade 2; G_3_, BOAS Grade 3. ^a,b, c, d^different letters indicate significant differences *p* < 0.05 between groups. ^1, 2, 3^different number indicate significant differences *p* < 0.05 between times.

#### 3.3.3 Muscular regions

T°*gracilis* showed that B_3_ across all BOAS grades exhibited temperatures between 31 °C and 35 °C. G_2_ recorded the highest increase at T_3_ (35.05 °C ± 0.49 °C), with statistically significant differences compared to B_1_, B_2_, and G_3_ (*p* < 0.0001).

For T°*femoralis* and T°*gastrocnemius*, B_3_ groups G_1_ and G_2_ presented higher maximum (Supplementary material Table S4) and mean temperatures (Table 2) at T_2_, T_3_, and T_4_. The highest values were recorded at T_3_ in G_2_, with a minimum of 33.27 °C ± 0.52 °C and a maximum of 33.66 °C ± 0.55 °C (*p* < 0.0001).

Biotypes B_1_ and B_2_ showed modest increases of 1 °C−1.5 °C across all three muscular regions at T_2_ and T*3*, returning to baseline values at T_4_.

### 3.4 Correlations between thermal windows and physical parameters

Highly significant correlations were identified between thermal windows and variables such as distance traveled and time. Additional factors, including age, weight, and body condition score, were also analyzed (Supplementary material: Tables S1–S15).

In B_1_, significant positive correlations were found between time and minimum, maximum, and mean temperatures across the seven facial and five appendicular thermal windows (*r*= 0.644 to 0.884, *p* < 0.001). Regions of interest including ${\text{T}}_{\text{caruncle}}^{{}^{\circ}}$, ${\text{T}}_{\text{uppereyelid}}^{{}^{\circ}}$, ${\text{T}}_{\text{lowereyelid}}^{{}^{\circ}}$, ${\text{T}}_{\text{upperlip}}^{{}^{\circ}}$, ${\text{T}}_{\text{lowerlip}}^{{}^{\circ}}$, ${\text{T}}_{\text{forelimbs}}^{{}^{\circ}}$, ${\text{T}}_{\text{hindlimbs},}^{{}^{\circ}}$ T°*f*_*emoralis*_, T°_*gastrocnemius*_, and T°_*gracilis*_ were highly correlated with each other (*r*= 0.92 to 0.99, *p* < 0.001).

In B_2_, significant positive correlations were observed between the time and minimum, maximum, and mean temperatures across all thermal windows (*r*= 0.753 to 0.884, *p* < 0.001), with strong intercorrelations between the facial and appendicular regions (*r*> 0.95, *p* < 0.001).

In B_3_, highly significant positive correlations were observed between time and thermal variables across all windows (*r*= 0.753 to 0.828, *p* < 0.001). Similar patterns were observed in subdivisions G_1_, G_2_, and G_3_, with facial regions showing strong correlations (*r*= 0.95 to 0.99, *p* < 0.001) and appendicular regions also demonstrating high correlations (r = 0.90 to 0.98, *p* < 0.001).

Conversely, correlations between age and both facial and appendicular thermal windows were not significant in any group (*r*= 0.17 to 0.33), similar to those for distance (*r*= 0.10 to 0.20) and time (*r*= 0.15 to 0.25). Only weight showed a highly significant correlation (*r*= 0.86 to 0.96).

## 4 Discussion

Infrared thermography is a technique that quantifies skin surface temperature by visualizing microcirculatory changes. Therefore, surface thermal variations measured by infrared thermography enable sensitive characterization of physiological responses (2). It is important to recognize that although body temperature continuously fluctuates due to various physiological mechanisms, physical activity induces stress responses and metabolic adaptations, in which animals mobilize muscle glycogen and fat as energy sources, activating anaerobic metabolism when maximal effort is required, as has been observed in species such as horses (21) and dogs (22).

For instance, Nold et al. (23) reported that the greyhound is the only breed capable of developing lactate concentrations of up to 47.2 mmol/l and a pH of 6.899, compared to horses (14.3 mEq/l) (24) and human athletes (13.9 mEq/l) (25). They also indicated that additional markers beyond lactate may be affected by prolonged exercise. For example, at different distances, increases in both temperature and potassium levels are observed, with values reported for 402 m (39.6 °C ± 0.2 °C and 5.1 ± 0.2 mEq/L), 503 m (38.8 °C ± 0.1 °C and 4.4 ± 0.1 mEq/L), and 704 m (38.7 °C ± 0.2 °C and 4.4 ± 0.1 mEq/L), respectively.

Similarly, Zaldívar-López et al. (26) reported that even within the same breed, such as Spanish greyhounds, performance differences may exist. In their study, 11 out of 17 blood parameters showed statistically significant differences between Spanish greyhounds and mixed breeds under basal conditions. Elevated concentration of bicarbonate, pCO_2_, total CO_2_, total hemoglobin, and oxygen content were observed in greyhounds, whereas pH, chloride concentration, and P50 were reduced. These values reflect the enhanced efficiency of oxygen transport in athletic greyhounds.

On the other hand, extreme physical conformation may also play a role in exercise performance and hyperthermia, as reported by Reyes-Sotelo et al. (18) in brachycephalic dogs, where a muzzle length below 38 mm was associated with a high risk of obstruction, particularly in BOAS grades 2 and 3. This condition resulted in oxygen saturation levels below 90 percent and maximum rectal temperatures of 39.23 °C ± 0.07 °C during a 1,000 m walk test in BOAS grade 3 dogs, without adequate recovery after 10 min (39.12 °C ± 0.05 °C), confirming impaired thermoregulation.

In contrast, in normocephalic dogs, Baker et al. (27) demonstrated that during exercise, cardiac output increased by 74 to 200 percent and carotid blood flow by up to 500 percent at ambient temperatures between 25 °C and 35 °C. These findings indicate that carotid blood flow is closely related to thermoregulatory demands, with increased flow directed toward evaporative surfaces such as the nose, mouth, and tongue. This highlights the influence of biotype, as dogs in group B3 exhibited higher facial temperatures at both baseline (T_1_) and recovery (T_4_).

The effect of biotype was further reflected in G_1_, G_2_, and G_3_ due to extreme facial morphometry. Brachycephalic dogs showed greater thermal accumulation in facial thermal windows, consistent with previous studies that have demonstrated reduced capacity for heat dissipation through respiration due to craniofacial conformation (5, 6). Severe brachycephaly leads to deformation of the upper airway tract and subsequent obstruction, as the soft tissues are not proportionally reduced relative to skull length (28) (Figure 7).

**Figure 7:**
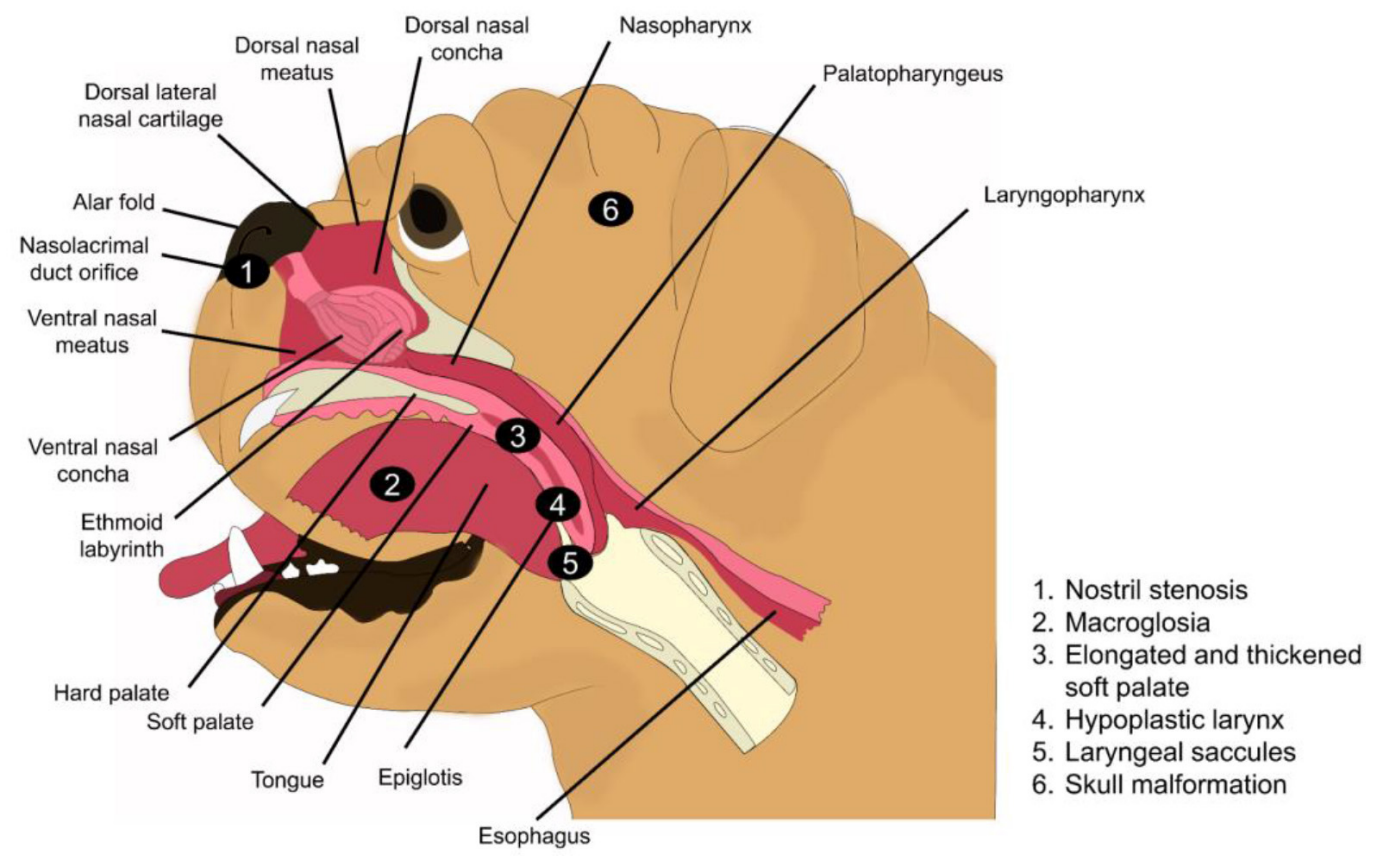
Upper airway tract and the abnormalities present in brachycephalic dogs. Original creation using information from Andrews et al. (20).

Consequently, these dogs exhibit difficult breathing accompanied by respiratory noise (29), sleep-disturbed breathing (30), sleep apnoea (31), and gastrointestinal disorders such as vomiting, regurgitation, and ptyalism. Additionally, many cases present with hiatal hernias and pyloric stenosis, and in severe cases, cyanosis, collapse, and death may occur (32, 33).

From a pathophysiological perspective of BOAS, the search for less invasive diagnostic approaches has led to the identification of the lacrimal caruncle as a reliable site for detecting thermal increases during stress events due to sympathetic activation detected by i infrared thermography (34). This region is highly vascularized and hairless, allowing accurate temperature measurement without interference from environmental conditions or coat color (35).

Regarding BOAS severity, G_2_ and G_3_ dogs exhibited pronounced facial thermal increases, slower recovery, and thermoregulatory instability. These findings are consistent with those reported by Lilja-Maula et al. (16), who demonstrated that BOAS severity correlates with reduced performance in submaximal exercise tests and increased respiratory load.

Importantly, the nostril status and degree of stenosis have traditionally been assessed as static parameters. However, the present study supports the concept of this anatomical region as a dynamic structure across different biotypes, showing substantial variability during exercising, sniffing, and panting, facilitating a higher flow rate, as previously described by Evans and de Lahunta (36) and Sharp et al. (37).

Soroko et al. (38) investigated the effect of high-intensity exercise on surface temperature in healthy beagle dogs using infrared thermography, evaluating multiple anatomical regions. They reported that temperature changes were most pronounced in the forelimbs and hindlimbs, with increases observed after trotting and persisting up to 15 min post-exercise. Peak temperatures were recorded after canter and following a second walking phase, indicating that greater muscular activity leads to increased heat production and subsequent superficial vasodilation.

Similarly, Vainionpää et al. (39) reported temperature increases of 0.6 °C to 2.1 °C in the *gastrocnemius muscle* of racing greyhounds following different running distances, associated with increased muscular blood flow to meet oxygen demand. Efficient heat dissipation was achieved through convection, conduction, evaporation, and panting, preventing hyperthermia.

These findings align with the present study, in which significant thermal increases following T_2_ and T_3_ were attributed to increased muscle metabolism, redistribution of blood flow, and activation of peripheral heat dissipation mechanisms (1, 40). This response has been described as biphasic, involving initial vasoconstriction followed by heat-dependent vasodilation as part of thermoregulatory adjustment (41).

Differential activation of the *gracilis, biceps femoralis*, and *gastrocnemius muscles* may explain appendicular thermal variations, as suggested by Deban et al. (42) in relation to gait and muscle activation. However, it should be considered that appendicular thermal readings may be influenced by hair density and type, as observed in glabrous skin, short hair, and long hair regions (43, 44).

Repac et al. (9) reported significant post-exercise increases in surface temperature of the *biceps femoris* and *gracilis muscles* following a 6-min walk test, suggesting that evaluating multiple regions along the muscle provides a more accurate assessment of vascular changes than single point measurements.

In the present study, increased heat stress in brachycephalic dogs, particularly in BOAS grade 3, likely triggered neuroendocrine responses involving adrenocorticotropic hormone release, followed by catecholamines and adrenal glucocorticoids, which regulate energy metabolism (45). Impaired heat dissipation in this group led to internal heat accumulation within the abdominal and thoracic cavities, similar to findings reported in swine (46, 47). This may explain the reduced values in appendicular windows observed in severe BOAS compared to grades 1 and 2, where, despite hyperthermia across all groups, only BOAS grade 3 exhibited peripheral tissue hypoxia, increased carbon dioxide levels, reduced partial pressure of oxygen, dyspnoea, and panting, suggesting enhanced glycolysis and lactate accumulation (48, 49) (Figure 8).

**Figure 8:**
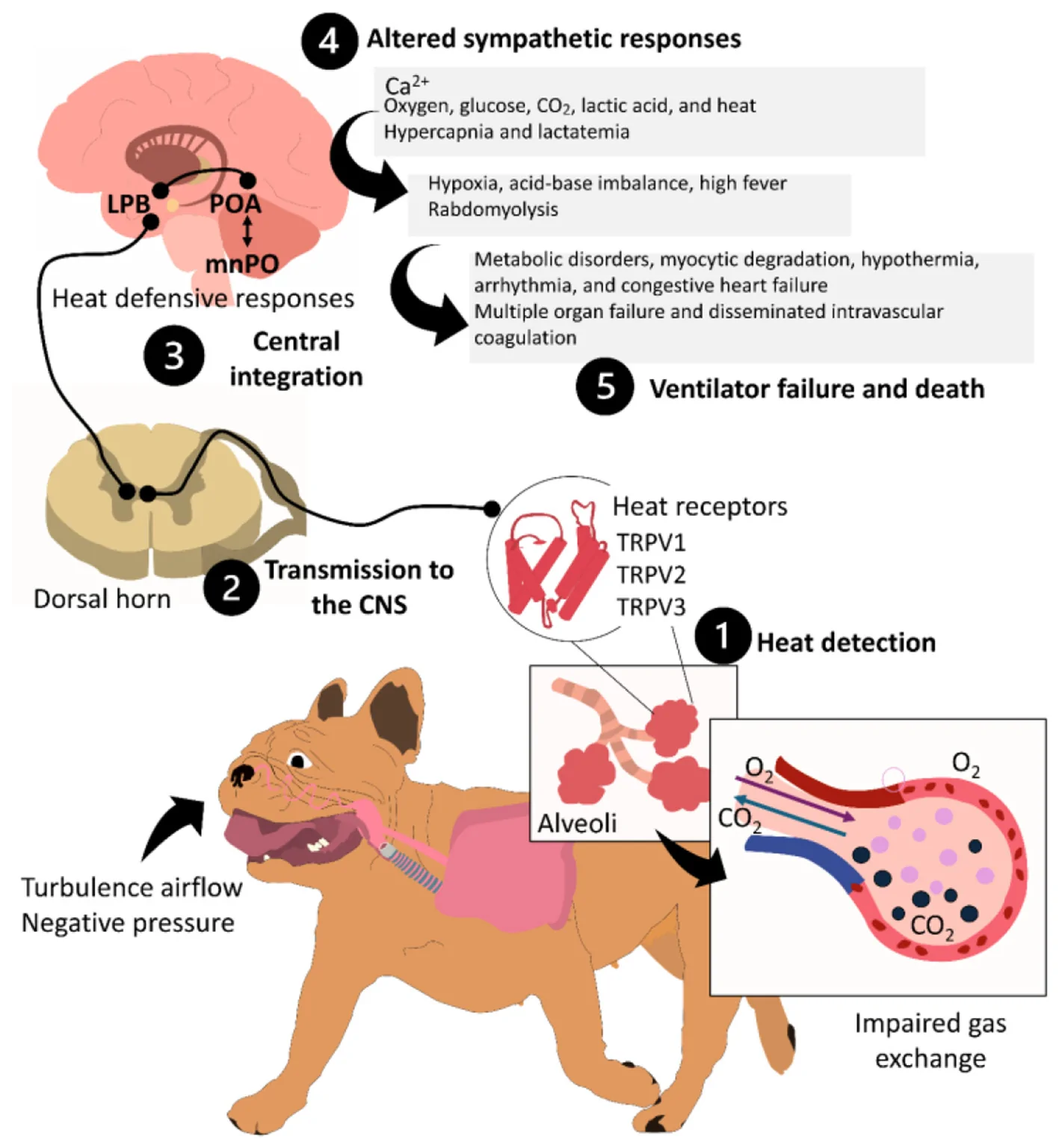
Exercise thermal response of the brachycephalic. The Autonomic Nervous System perceives the increase in heat through its thermoreceptors and sends these signals to POA. This area, in response, sends electrical discharges to the heat losss centers, wich in turn activate descending vasomotors and sudomotor nerves. These nerves reach the target sites, triggering defensive responses to heat. Similary, CO_2_ is detected by baroreceptors and chemoreceptors, which regulate the respiratory control center, working together to increae gas exchange. However, in brachycephalic dogs, due to anatomically restrictive processes specially in breathing, acid-base imbalance, hypoxia, and restricted appendicular vasodilatation occur. LPB, lateral parabrachial nucleus; MnPO, median preoptic area; POA, preoptical area.

The positive correlation between thermal windows and physical performance variables suggests that infrared thermography may serve as a complementary biomarker for evaluating exercise tolerance in brachycephalic dogs, alongside the BOAS functional grading system and plethysmography (19).

These results reinforce the utility of IRT in the functional assessment of brachycephalic dogs and its potential for detecting early alterations associated with BOAS. Furthermore, both standardized exercise tests provide valuable tools for evaluating functional capacity without confounding effects of additional cardiorespiratory or muscular pathologies (10, 15).

These findings are consistent with the established role of cutaneous blood flow in mammalian thermoregulation. During exercise, increased metabolic heat engages thermoregulatory heat-loss mechanisms, including peripheral vasodilation and evaporative cooling, which can produce measurable changes in skin surface temperature. Infrared thermography may therefore provide indirect information about these thermoregulatory adjustments through changes in superficial microcirculation and temperature within thermal windows. However, interpretation should consider the anatomical region evaluated, sympathetic vascular modulation, and biological and environmental factors that may influence surface temperature measurements. IRT should therefore be regarded as a complementary tool for assessing thermoregulatory responses rather than as an isolated measure of core temperature or physiological impairment (50–52).

## 5 Conclusions

This study demonstrates that IRT enables the identification of facial and appendicular thermal variations associated with exercise through two standardized tests, which may contribute to the elucidation of physiological responses associated with different degrees of BOAS. This approach allows precise characterization of thermoregulatory responses and functional airway compromise resulting from the extreme facial conformation of the brachycephalic biotype. In this context, this biotype exhibited greater thermal increases in both the facial and appendicular thermal windows, resulting in slower post exercise recovery, confirming its limited capacity for heat dissipation. This limitation may be associated with ventilatory alterations derived from its anatomical conformation.

Overall, these findings further support the value of multimodal diagnostic approaches to evaluate aerobic capacity, superficial microcirculation, and respiratory function, ultimately contributing to improved diagnosis, monitoring, and clinical management of brachycephalic dogs.

## 6 Study limitations

One of the main limitations of this study was the lack of standardized environmental conditions, which may have influenced thermal responses. However, this setting was intentionally selected to evaluate clinical applicability under variable temperature and humidity conditions.

The study was conducted in Mexico City, at an altitude of 2,240 m above sea level. Therefore, caution is warranted when extrapolating these findings to populations living at lower altitudes, as altitude-related differences in oxygen availability and physiological demand may influence the measured responses.

Coat type was not evaluated and should be considered in future studies as a potential source of variability.

Finally, the limited variability within the brachycephalic biotype reflected the availability of animals in the study region and the limited participation of breeders and owners of English and French Bulldogs.

## Data Availability

The original contributions presented in the study are included in the article/Supplementary material, further inquiries can be directed to the corresponding author/s.
