# Supplementary material for: Thermal response to moderate exercise in dogs of different biotypes and brachycephalic obstructive airway syndrome severity assessed using infrared thermography
Source: Front Vet Sci. 2026 Oct 26;13:1975908. doi: 10.3389/fvets.2026.1975908 (PMC13745323; doi:10.3389/fvets.2026.1975908)
Supplement: Supplementary file 1 [file Table_1.docx]

Table S1. Correlation matrix of the dolichocephalic group “B1”. Minimum temperature of thermal windows and physical parameters

| Dolichocephalic (Minimum) | Distance | Time | Age | Weight | T° Caruncle | T°  Upper eyelid | T° Lower eyelid | T° Upper lip_ | T° Lower lip_ | T°  Mas | T°  nostril | T°  Flimbs | T° Hlinbs | T° *Gracilis* | | T° *Femoralis* | | T° *Gastocnemio* | | |
| --- | --- | --- | --- | --- | --- | --- | --- | --- | --- | --- | --- | --- | --- | --- | --- | --- | --- | --- | --- | --- |
| Distance | 1 |  |  |  |  |  |  |  |  |  |  |  |  | |  | |  | |  |  |
| Time | 0.588 p<0.001 | 1 |  |  |  |  |  |  |  |  |  |  |  | |  | |  | |  |  |
| Age | 0.052 p=0.650 | 0.059 p=0.601 | 1 |  |  |  |  |  |  |  |  |  |  | |  | |  | |  |  |
| Weight | 0.058 p=0.607 | 0.062 p=0.585 | 0.773 p<0.001 | 1 |  |  |  |  |  |  |  |  |  | |  | |  | |  |  |
| T° Caruncle | 0.304 p=0.006 | 0.876 p<0.001 | 0.297 p=0.007 | 0.251 p=0.025 | 1 |  |  |  |  |  |  |  |  | |  | |  | |  |  |
| T° upper eyelid | 0.335 p=0.002 | 0.877 p<0.001 | 0.298 p=0.007 | 0.248 p=0.027 | 0.990 p<0.001 | 1 |  |  |  |  |  |  |  | |  | |  | |  |  |
| T° Lower eyelid | 0.363 p=0.001 | 0.883 p<0.001 | 0.282 p=0.011 | 0.249 p=0.026 | 0.990 p<0.001 | 0.996 p<0.001 | 1 |  |  |  |  |  |  | |  | |  | |  |  |
| T° Upper lip | 0.282 p=0.011 | 0.843 p<0.001 | 0.320 p=0.004 | 0.268 p=0.016 | 0.981 p<0.001 | 0.981 p<0.001 | 0.979 p<0.001 | 1 |  |  |  |  |  | |  | |  | |  |  |
| T° Lower lip | 0.228 p=0.042 | 0.866 p<0.001 | 0.302 p=0.007 | 0.254 p=0.023 | 0.986 p<0.001 | 0.976 p<0.001 | 0.969 p<0.001 | 0.977 p<0.001 | 1 |  |  |  |  | |  | |  | |  |  |
| T° Mas | 0.308 p=0.005 | 0.879 p<0.001 | 0.286 p=0.010 | 0.246 p=0.028 | 0.997 p<0.001 | 0.993 p<0.001 | 0.993 p<0.001 | 0.980 p<0.001 | 0.983 p<0.001 | 1 |  |  |  | |  | |  | |  |  |
| T° nostril | 0.128 p=0.258 | 0.820 p<0.001 | 0.303 p=0.006 | 0.264 p=0.018 | 0.944 p<0.001 | 0.918 p<0.001 | 0.907 p<0.001 | 0.929 p<0.001 | 0.979 p<0.001 | 0.936 p<0.001 | 1 |  |  | |  | |  | |  |  |
| T° Flimbs | 0.266 p=0.018 | 0.886 p<0.001 | 0.242 p=0.031 | 0.167 p=0.142 | 0.981 p<0.001 | 0.967 p<0.001 | 0.961 p<0.001 | 0.971 p<0.001 | 0.994 p<0.001 | 0.978 p<0.001 | 0.979 p<0.001 | 1 |  | |  | |  | |  |  |
| T° Hlimbs | 0.301 p=0.007 | 0.905 p<0.001 | 0.252 p=0.024 | 0.223 p=0.047 | 0.985 p<0.001 | 0.974 p<0.001 | 0.972 p<0.001 | 0.970 p<0.001 | 0.991 p<0.001 | 0.981 p<0.001 | 0.963 p<0.001 | 0.994 p<0.001 | 1 | |  | |  | |  |  |
| T° *Gracilis* | 0.249 p=0.026 | 0.897 p<0.001 | 0.233 p=0.038 | 0.214 p=0.057 | 0.968 p<0.001 | 0.949 p<0.001 | 0.946 p<0.001 | 0.946 p<0.001 | 0.985 p<0.001 | 0.963 p<0.001 | 0.973 p<0.001 | 0.990 p<0.001 | 0.993 p<0.001 | | 1 | |  | |  |  |
| T° *Femoralis* | 0.24 p=0.032 | 0.898 p<0.001 | 0.227 p=0.043 | 0.200 p=0.075 | 0.952 p<0.001 | 0.935 p<0.001 | 0.931 p<0.001 | 0.933 p<0.001 | 0.975 p<0.001 | 0.946 p<0.001 | 0.972 p<0.001 | 0.982 p<0.001 | 0.985 p<0.001 | | 0.993 p<0.001 | | 1 | |  |  |
| T° *Gastocnemio* | 0.197 p=0.080 | 0.880 p<0.001 | 0.224 p=0.046 | 0.201 p=0.073 | 0.935 p<0.001 | 0.918 p<0.001 | 0.911 p<0.001 | 0.923 p<0.001 | 0.967 p<0.001 | 0.930 p<0.001 | 0.971 p<0.001 | 0.975 p<0.001 | 0.974 p<0.001 | | 0.987 p<0.001 | | 0.995 p<0.001 | | 1 |  |

Table S2. Correlation matrix of the dolichocephalic group “B1”. Maximum temperature of thermal windows and physical parameters.

| Dolichocephalic  (Maximum) | Distance | Time | Age | Weight | T° Caruncle | T°  Upper eyelid | T°  Lower eyelid | T° Upper lip | T° Lower lip | T°  Mas | T°  nostril | T°  Flimbs | T°  Hlimbs | T°  *Gracilis* | T°  *Femoralis* | T° *Gastocnemio* |
| --- | --- | --- | --- | --- | --- | --- | --- | --- | --- | --- | --- | --- | --- | --- | --- | --- |
| Distance | 1 |  |  |  |  |  |  |  |  |  |  |  |  |  |  |  |
| Time | 0.588 p<0.001 | 1 |  |  |  |  |  |  |  |  |  |  |  |  |  |  |
| Age | 0.052 p=0.650 | 0.059 p=0.601 | 1 |  |  |  |  |  |  |  |  |  |  |  |  |  |
| Weight | 0.058 p=0.607 | 0.062 p=0.585 | 0.773 p<0.001 | 1 |  |  |  |  |  |  |  |  |  |  |  |  |
| T° Caruncle | 0.299 p=0.007 | 0.828 p<0.001 | 0.33 p=0.003 | 0.280 p=0.012 | 1 |  |  |  |  |  |  |  |  |  |  |  |
| T° Upper eyelid | 0.294 p=0.008 | 0.844 p<0.001 | 0.316 p=0.004 | 0.277 p=0.013 | 0.996 p<0.001 | 1 |  |  |  |  |  |  |  |  |  |  |
| T° Lower eyelid | 0.303 p=0.006 | 0.864 p<0.001 | 0.304 p=0.006 | 0.255 p=0.023 | 0.982 p<0.001 | 0.988 p<0.001 | 1 |  |  |  |  |  |  |  |  |  |
| T° Upper lip | 0.254 p=0.023 | 0.860 p<0.001 | 0.310 p=0.005 | 0.261 p=0.020 | 0.979 p<0.001 | 0.988 p<0.001 | 0.990 p<0.001 | 1 |  |  |  |  |  |  |  |  |
| T° Lower lip | 0.238 p=0.034 | 0.845 p<0.001 | 0.316 p=0.004 | 0.261 p=0.020 | 0.985 p<0.001 | 0.991 p<0.001 | 0.985 p<0.001 | 0.992 p<0.001 | 1 |  |  |  |  |  |  |  |
| T° Mas | 0.347 p=0.002 | 0.869 p<0.001 | 0.284 p=0.011 | 0.239 p=0.033 | 0.985 p<0.001 | 0.990 p<0.001 | 0.980 p<0.001 | 0.982 p<0.001 | 0.981 p<0.001 | 1 |  |  |  |  |  |  |
| T° nostril | 0.149 p=0.186 | 0.836 p<0.001 | 0.283 p=0.011 | 0.235 p=0.036 | 0.942 p<0.001 | 0.955 p<0.001 | 0.967 p<0.001 | 0.982 p<0.001 | 0.972 p<0.001 | 0.939 p<0.001 | 1 |  |  |  |  |  |
| T° Flimbs | 0.236 p=0.037 | 0.812 p<0.001 | 0.320 p=0.004 | 0.232 p=0.040 | 0.979 p<0.001 | 0.983 p<0.001 | 0.973 p<0.001 | 0.982 p<0.001 | 0.987 p<0.001 | 0.982 p<0.001 | 0.947 p<0.001} | 1 |  |  |  |  |
| T° Hlimbs | 0.320 p=0.004 | 0.904 p<0.001 | 0.245 p=0.028 | 0.223 p=0.046 | 0.952 p<0.001 | 0.966 p<0.001 | 0.970 p<0.001 | 0.981 p<0.001 | 0.977 p<0.001 | 0.973 p<0.001 | 0.959 p<0.001 | 0.958 p<0.001 | 1 |  |  |  |
| T° *Gracilis* | 0.281 p=0.011 | 0.881 p<0.001 | 0.275 p=0.014 | 0.231 p=0.040 | 0.969 p<0.001 | 0.978 p<0.001 | 0.980 p<0.001 | 0.988 p<0.001 | 0.987 p<0.001 | 0.976 p<0.001 | 0.975 p<0.001 | 0.965 p<0.001 | 0.991 p<0.001 | 1 |  |  |
| T° *Femoralis* | 0.262 p=0.019 | 0.901 p<0.001 | 0.221 p=0.049 | 0.192 p=0.088 | 0.919 p<0.001 | 0.936 p<0.001 | 0.961 p<0.001 | 0.970 p<0.001 | 0.955 p<0.001 | 0.927 p<0.001 | 0.980 p<0.001 | 0.921 p<0.001 | 0.967 p<0.001 | 0.968 p<0.001 | 1 |  |
| T° *Gastocnemio* | 0.304 p=0.006 | 0.895 p<0.001 | 0.274 p=0.014 | 0.230 p=0.040 | 0.946 p<0.001 | 0.960 p<0.001 | 0.978 p<0.001 | 0.984 p<0.001 | 0.971 p<0.001 | 0.957 p<0.001 | 0.980 p<0.001 | 0.941 p<0.001 | 0.984 p<0.001 | 0.988 p<0.001 | 0.987 p<0.001 | 1 |

Table S3. Correlation matrix of the dolicocephalic group “B1”. Average temperature of thermal windows and physical parameters.

| Dolichocephalic  (AVERAGE) | Distance | Time | Age | Weight | T°  Caruncle_ | T°  Upper eyelid | T°  Lower eyelid | T°  Upper lip | T°  Lower lip | T°  Mas | T°  nostril | T°  Flimbs | T°  Hlimbs | T°  *Gracilis* | T°  *Femoralis* | T°  *Gastocnemio* |
| --- | --- | --- | --- | --- | --- | --- | --- | --- | --- | --- | --- | --- | --- | --- | --- | --- |
| Distance | 1 |  |  |  |  |  |  |  |  |  |  |  |  |  |  |  |
| Time | 0.588 p<0.001 | 1 |  |  |  |  |  |  |  |  |  |  |  |  |  |  |
| Age | 0.052 p=0.650 | 0.059 p=0.601 | 1 |  |  |  |  |  |  |  |  |  |  |  |  |  |
| Weight | 0.058 p=0.607 | 0.062 p=0.585 | 0.773 p<0.001 | 1 |  |  |  |  |  |  |  |  |  |  |  |  |
| T°Caruncle | 0.280 p=0.012 | 0.827 p<0.001 | 0.339 p=0.002 | 0.285 p=0.010 | 1 |  |  |  |  |  |  |  |  |  |  |  |
| T°Upper eyelid | 0.311 p=0.005 | 0.858 p<0.001 | 0.317 p=0.004 | 0.270 p=0.015 | 0.987 p<0.001 | 1 |  |  |  |  |  |  |  |  |  |  |
| T°Lower eyelid | 0.341 p=0.002 | 0.884 p<0.001 | 0.291 p=0.009 | 0.252 p=0.024 | 0.985 p<0.001 | 0.986 p<0.001 | 1 |  |  |  |  |  |  |  |  |  |
| T°Upper lip | 0.286 p=0.010 | 0.855 p<0.001 | 0.325 p=0.003 | 0.270 p=0.015 | 0.985 p<0.001 | 0.992 p<0.001 | 0.990 p<0.001 | 1 |  |  |  |  |  |  |  |  |
| T°Lower lip | 0.197 p=0.080 | 0.644 p<0.001 | 0.339 p=0.002 | 0.258 p=0.021 | 0.847 p<0.001 | 0.846 p<0.001 | 0.838 p<0.001 | 0.871 p<0.001 | 1 |  |  |  |  |  |  |  |
| T°Mas | 0.291 p=0.009 | 0.867 p<0.001 | 0.289 p=0.009 | 0.246 p=0.028 | 0.982 p<0.001 | 0.991 p<0.001 | 0.993 p<0.001 | 0.994 p<0.001 | 0.852 p<0.001 | 1 |  |  |  |  |  |  |
| T°noatril | 0.097 p=0.391 | 0.808 p<0.001 | 0.295 p=0.008 | 0.243 p=0.030 | 0.917 p<0.001 | 0.915 p<0.001 | 0.926 p<0.001 | 0.938 p<0.001 | 0.774 p<0.001 | 0.935 p<0.001 | 1 |  |  |  |  |  |
| T°Flimbs | 0.253 p=0.025 | 0.833 p<0.001 | 0.298 p=0.008 | 0.206 p=0.070 | 0.981 p<0.001 | 0.985 p<0.001 | 0.980 p<0.001 | 0.993 p<0.001 | 0.870 p<0.001 | 0.988 p<0.001 | 0.949 p<0.001 | 1 |  |  |  |  |
| T°Hlimbs | 0.316 p=0.004 | 0.904 p<0.001 | 0.262 p=0.019 | 0.223 p=0.047 | 0.973 p<0.001 | 0.979 p<0.001 | 0.991 p<0.001 | 0.988 p<0.001 | 0.821 p<0.001 | 0.990 p<0.001 | 0.951 p<0.001 | 0.979 p<0.001 | 1 |  |  |  |
| T°*Gracilis* | 0.274 p=0.014 | 0.895 p<0.001 | 0.254 p=0.023 | 0.226 p=0.044 | 0.955 p<0.001 | 0.955 p<0.001 | 0.976 p<0.001 | 0.969 p<0.001 | 0.783 p<0.001 | 0.972 p<0.001 | 0.971 p<0.001 | 0.963 p<0.001 | 0.990 p<0.001 | 1 |  |  |
| T°*Femoralis* | 0.254 p=0.023 | 0.894 p<0.001 | 0.224 p=0.045 | 0.200 p=0.075 | 0.920 p<0.001 | 0.932 p<0.001 | 0.943 p<0.001 | 0.943 p<0.001 | 0.760 p<0.001 | 0.941 p<0.001 | 0.964 p<0.001 | 0.939 p<0.001 | 0.969 p<0.001 | 0.983 p<0.001 | 1 |  |
| T°*Gastocnemio* | 0.253 p=0.024 | 0.885 p<0.001 | 0.265 p=0.017 | 0.248 p=0.026 | 0.936 p<0.001 | 0.943 p<0.001 | 0.959 p<0.001 | 0.959 p<0.001 | 0.789 p<0.001 | 0.955 p<0.001 | 0.970 p<0.001 | 0.955 p<0.001 | 0.978 p<0.001 | 0.990 p<0.001 | 0.992 p<0.001 | 1 |

Table S4. Correlation matrix mesocephalic group “B2”. Minimum temperature of thermal windows and physical parameters.

| Mesocephalic  (Minimum) | Distance | Time | Age | Weight | T°  Caruncle | T°  Upper eyelid | T°  Lower eyelid | T°  Upper lip | T°  Lower lip | T°  Mas | T°  nostril | T°  Flimbs | T°  Hlimbs | T°  *Gracilis* | T°  *Femoralis* | T°  *Gastocnemio* |
| --- | --- | --- | --- | --- | --- | --- | --- | --- | --- | --- | --- | --- | --- | --- | --- | --- |
| Distance | 1 |  |  |  |  |  |  |  |  |  |  |  |  |  |  |  |
| Time | 0.614 p<0.001 | 1 |  |  |  |  |  |  |  |  |  |  |  |  |  |  |
| Age | 0.107 p=0.345 | 0.145 p=0.200 | 1 |  |  |  |  |  |  |  |  |  |  |  |  |  |
| Weight | 0.092 p=0.417 | 0.142 p=0.210 | 0.889 p<0.001 | 1 |  |  |  |  |  |  |  |  |  |  |  |  |
| T°Caruncle | 0.269 p=0.016 | 0.817 p<0.001 | 0.283 p=0.011 | 0.270 p=0.015 | 1 |  |  |  |  |  |  |  |  |  |  |  |
| T°Upper eyelid | 0.324 p=0.003 | 0.828 p<0.001 | 0.258 p=0.021 | 0.255 p=0.022 | 0.992 p<0.001 | 1 |  |  |  |  |  |  |  |  |  |  |
| T°Lower eyelid | 0.374 p=0.001 | 0.819 p<0.001 | 0.254 p=0.023 | 0.230 p=0.040 | 0.970 p<0.001 | 0.982 p<0.001 | 1 |  |  |  |  |  |  |  |  |  |
| T°Upper lip | 0.348 p=0.002 | 0.851 p<0.001 | 0.253 p=0.024 | 0.240 p=0.032 | 0.994 p<0.001 | 0.993 p<0.001 | 0.982 p<0.001 | 1 |  |  |  |  |  |  |  |  |
| T°Lower lip | 0.310 p=0.005 | 0.813 p<0.001 | 0.273 p=0.014 | 0.252 p=0.024 | 0.991 p<0.001 | 0.993 p<0.001 | 0.987 p<0.001 | 0.992 p<0.001 | 1 |  |  |  |  |  |  |  |
| T°Mas | 0.264 p=0.018 | 0.819 p<0.001 | 0.289 p=0.009 | 0.279 p=0.012 | 0.991 p<0.001 | 0.982 p<0.001 | 0.954 p<0.001 | 0.982 p<0.001 | 0.979 p<0.001 | 1 |  |  |  |  |  |  |
| T°nostril | 0.171 p=0.130 | 0.766 p<0.001 | 0.285 p=0.010 | 0.276 p=0.013 | 0.976 p<0.001 | 0.959 p<0.001 | 0.920 p<0.001 | 0.959 p<0.001 | 0.956 p<0.001 | 0.973 p<0.001 | 1 |  |  |  |  |  |
| T°Flimbs | 0.224 p=0.045 | 0.808 p<0.001 | 0.282 p=0.011 | 0.281 p=0.011 | 0.993 p<0.001 | 0.982 p<0.001 | 0.951 p<0.001 | 0.982 p<0.001 | 0.978 p<0.001 | 0.992 p<0.001 | 0.990 p<0.001 | 1 |  |  |  |  |
| T°Hlimbs | 0.158 p=0.161 | 0.773 p<0.001 | 0.289 p=0.009 | 0.287 p=0.010 | 0.986 p<0.001 | 0.970 p<0.001 | 0.931 p<0.001 | 0.968 p<0.001 | 0.965 p<0.001 | 0.982 p<0.001 | 0.992 p<0.001 | 0.995 p<0.001 | 1 |  |  |  |
| T°*Gracilis* | 0.066 p=0.560 | 0.727 p<0.001 | 0.296 p=0.008 | 0.291 p=0.009 | 0.948 p<0.001 | 0.919 p<0.001 | 0.876 p<0.001 | 0.920 p<0.001 | 0.920 p<0.001 | 0.954 p<0.001 | 0.982 p<0.001 | 0.970 p<0.001 | 0.979 p<0.001 | 1 |  |  |
| T°*Femoralis* | 0.100 p=0.378 | 0.738 p<0.001 | 0.297 p=0.007 | 0.287 p=0.010 | 0.968 p<0.001 | 0.946 p<0.001 | 0.915 p<0.001 | 0.945 p<0.001 | 0.948 p<0.001 | 0.966 p<0.001 | 0.987 p<0.001 | 0.981 p<0.001 | 0.988 p<0.001 | 0.990 p<0.001 | 1 |  |
| T°*Gastocnemio* | 0.015 p=0.893 | 0.679 p<0.001 | 0.301 p=0.007 | 0.291 p=0.009 | 0.934 p<0.001 | 0.904 p<0.001 | 0.858 p<0.001 | 0.901 p<0.001 | 0.906 p<0.001 | 0.933 p<0.001 | 0.977 p<0.001 | 0.958 p<0.001 | 0.973 p<0.001 | 0.992 p<0.001 | 0.986 p<0.001 | 1 |

Table S5. Correlation matrix mesocephalic group “B2”. Maximum temperature of thermal windows and physical parameters.

| Mesocephalic  (Maximum) | Distance | Time | Age | Weight | T°  Caruncle | T°  Upper eyelid | T°L  ower eyelid | T°  Upper lip | T°  Lower lip | T°  Mas | T°  nostril | T°  Flimbs | T°  Hlimbs | T°  *Gracilis* | T°  *Femoralis* | T°  *Gastocnemio* |
| --- | --- | --- | --- | --- | --- | --- | --- | --- | --- | --- | --- | --- | --- | --- | --- | --- |
| Distance | 1 |  |  |  |  |  |  |  |  |  |  |  |  |  |  |  |
| Time | 0.614 p<0.001 | 1 |  |  |  |  |  |  |  |  |  |  |  |  |  |  |
| Age | 0.107 p=0.345 | 0.145 p=0.200 | 1 |  |  |  |  |  |  |  |  |  |  |  |  |  |
| Weight | 0.092 p=0.417 | 0.142 p=0.210 | 0.889 p<0.001 | 1 |  |  |  |  |  |  |  |  |  |  |  |  |
| T°Caruncle | 0.263 p=0.018 | 0.663 p<0.001 | 0.364 p=0.001 | 0.318 p=0.004 | 1 |  |  |  |  |  |  |  |  |  |  |  |
| T°Upper eyelid | 0.301 p=0.007 | 0.744 p<0.001 | 0.322 p=0.004 | 0.301 p=0.007 | 0.974 p<0.001 | 1 |  |  |  |  |  |  |  |  |  |  |
| L T°ower eyelid | 0.295 p=0.008 | 0.690 p<0.001 | 0.356 p=0.001 | 0.333 p=0.003 | 0.973 p<0.001 | 0.977 p<0.001 | 1 |  |  |  |  |  |  |  |  |  |
| T°Upper lip | 0.308 p=0.005 | 0.788 p<0.001 | 0.329 p=0.003 | 0.305 p=0.006 | 0.955 p<0.001 | 0.989 p<0.001 | 0.963 p<0.001 | 1 |  |  |  |  |  |  |  |  |
| T°Lower lip | 0.271 p=0.015 | 0.754 p<0.001 | 0.334 p=0.002 | 0.315 p=0.004 | 0.963 p<0.001 | 0.981 p<0.001 | 0.956 p<0.001 | 0.984 p<0.001} | 1 |  |  |  |  |  |  |  |
| T°Mas | 0.323 p=0.003 | 0.821 p<0.001 | 0.259 p=0.020 | 0.258 p=0.021 | 0.900 p<0.001 | 0.968 p<0.001 | 0.932 p<0.001 | 0.978 p<0.001 | 0.961 p<0.001 | 1 |  |  |  |  |  |  |
| T°nostril | 0.309 p=0.005 | 0.815 p<0.001 | 0.278 p=0.012 | 0.279 p=0.012 | 0.906 p<0.001 | 0.970 p<0.001 | 0.934 p<0.001 | 0.983 p<0.001 | 0.970 p<0.001 | 0.992 p<0.001 | 1 |  |  |  |  |  |
| T°Flimbs | 0.263 p=0.018 | 0.815 p<0.001 | 0.300 p=0.007 | 0.295 p=0.008 | 0.887 p<0.001 | 0.954 p<0.001 | 0.913 p<0.001 | 0.979 p<0.001 | 0.958 p<0.001 | 0.985 p<0.001 | 0.991 p<0.001 | 1 |  |  |  |  |
| T°Hlimbs | 0.212 p=0.059 | 0.803 p<0.001 | 0.298 p=0.007 | 0.297 p=0.008 | 0.899 p<0.001 | 0.956 p<0.001 | 0.912 p<0.001 | 0.977 p<0.001 | 0.962 p<0.001 | 0.981 p<0.001 | 0.984 p<0.001 | 0.991 p<0.001 | 1 |  |  |  |
| T°*Gracilis* | 0.169 p=0.133 | 0.760 p<0.001 | 0.314 p=0.005 | 0.297 p=0.007 | 0.897 p<0.001 | 0.951 p<0.001 | 0.911 p<0.001 | 0.973 p<0.001 | 0.962 p<0.001 | 0.971 p<0.001 | 0.981 p<0.001 | 0.988 p<0.001 | 0.989 p<0.001 | 1 |  |  |
| T°*Femoralis* | 0.110 p=0.334 | 0.753 p<0.001 | 0.301 p=0.007 | 0.287 p=0.010 | 0.852 p<0.001 | 0.915 p<0.001 | 0.862 p<0.001 | 0.947 p<0.001 | 0.935 p<0.001 | 0.950 p<0.001 | 0.960 p<0.001 | 0.979 p<0.001 | 0.984 p<0.001 | 0.991 p<0.001 | 1 |  |
| T°*Gastocnemio* | 0.064 p=0.571 | 0.740 p<0.001 | 0.285 p=0.010 | 0.280 p=0.012 | 0.828 p<0.001 | 0.889 p<0.001 | 0.834 p<0.001 | 0.926 p<0.001 | 0.918 p<0.001 | 0.930 p<0.001 | 0.942 p<0.001 | 0.966 p<0.001 | 0.973 p<0.001 | 0.981 p<0.001 | 0.994 p<0.001 | 1 |

Table S6. Correlation matrix mesocephalic group “B2”. Average temperature of thermal windows and physical paramtrers.

| Mesocephalic  (AVERAGE) | Distance | Time | Age | Weight | T°  Caruncle | T°  Upper eyelid | T°  Lower eyelid | T°  Upper lip | T°  Lower lip | T°  Mas | T°  nostril | T°  Flimbs | T°  Hlimbs | T°  *Gracilis* | T°  *Femoralis* | T°  *Gastocnemio* |
| --- | --- | --- | --- | --- | --- | --- | --- | --- | --- | --- | --- | --- | --- | --- | --- | --- |
| Distance | 1 |  |  |  |  |  |  |  |  |  |  |  |  |  |  |  |
| Time | 0.614 p<0.001 | 1 |  |  |  |  |  |  |  |  |  |  |  |  |  |  |
| Age | 0.107 p=0.345 | 0.145 p=0.200 | 1 |  |  |  |  |  |  |  |  |  |  |  |  |  |
| Weight | 0.092 p=0.417 | 0.142 p=0.210 | 0.889 p<0.001 | 1 |  |  |  |  |  |  |  |  |  |  |  |  |
| T°Caruncle | 0.329 p=0.003 | 0.780 p<0.001 | 0.309 p=0.005 | 0.296 p=0.008 | 1 |  |  |  |  |  |  |  |  |  |  |  |
| T°Upper eyelid | 0.344 p=0.002 | 0.795 p<0.001 | 0.308 p=0.005 | 0.290 p=0.009 | 0.988 p<0.001 | 1 |  |  |  |  |  |  |  |  |  |  |
| T°Lower eyelid | 0.342 p=0.002 | 0.738 p<0.001 | 0.342 p=0.002 | 0.319 p=0.004 | 0.983 p<0.001 | 0.982 p<0.001 | 1 |  |  |  |  |  |  |  |  |  |
| T°Upper lip | 0.353 p=0.001 | 0.837 p<0.001 | 0.267 p=0.017 | 0.258 p=0.021 | 0.983 p<0.001 | 0.990 p<0.001 | 0.960 p<0.001 | 1 |  |  |  |  |  |  |  |  |
| T°Lower lip | 0.270 p=0.016 | 0.759 p<0.001 | 0.316 p=0.004 | 0.297 p=0.008 | 0.993 p<0.001 | 0.989 p<0.001 | 0.986 p<0.001 | 0.981 p<0.001 | 1 |  |  |  |  |  |  |  |
| T°Mas | 0.310 p=0.005 | 0.833 p<0.001 | 0.256 p=0.022 | 0.240 p=0.032 | 0.974 p<0.001 | 0.985 p<0.001 | 0.946 p<0.001 | 0.994 p<0.001 | 0.974 p<0.001 | 1 |  |  |  |  |  |  |
| T°nostril | 0.230 p=0.040 | 0.819 p<0.001 | 0.248 p=0.027 | 0.242 p=0.030 | 0.933 p<0.001 | 0.952 p<0.001 | 0.890 p<0.001 | 0.970 p<0.001 | 0.937 p<0.001 | 0.981 p<0.001 | 1 |  |  |  |  |  |
| T°Flimbs | 0.229 p=0.041 | 0.804 p<0.001 | 0.293 p=0.008 | 0.282 p=0.011 | 0.960 p<0.001 | 0.972 p<0.001 | 0.928 p<0.001 | 0.980 p<0.001 | 0.965 p<0.001 | 0.988 p<0.001 | 0.992 p<0.001 | 1 |  |  |  |  |
| T°Hlimbs | 0.165 p=0.143 | 0.787 p<0.001 | 0.263 p=0.018 | 0.268 p=0.016 | 0.936 p<0.001 | 0.953 p<0.001 | 0.900 p<0.001 | 0.965 p<0.001 | 0.946 p<0.001 | 0.978 p<0.001 | 0.993 p<0.001 | 0.992 p<0.001 | 1 |  |  |  |
| T°*Gracilis* | 0.121 p=0.286 | 0.766 p<0.001 | 0.286 p=0.010 | 0.285 p=0.010 | 0.919 p<0.001 | 0.926 p<0.001 | 0.872 p<0.001 | 0.942 p<0.001 | 0.929 p<0.001 | 0.958 p<0.001 | 0.982 p<0.001 | 0.984 p<0.001 | 0.989 p<0.001 | 1 |  |  |
| T°*Femoralis* | 0.103 p=0.364 | 0.749 p<0.001 | 0.294 p=0.008 | 0.289 p=0.009 | 0.901 p<0.001 | 0.916 p<0.001 | 0.855 p<0.001 | 0.931 p<0.001 | 0.913 p<0.001 | 0.949 p<0.001 | 0.981 p<0.001 | 0.979 p<0.001 | 0.988 p<0.001 | 0.995 p<0.001 | 1 |  |
| T°*Gastocnemio* | -0.018 p=0.873 | 0.675 p<0.001 | 0.288 p=0.010 | 0.277 p=0.013 | 0.846 p<0.001 | 0.861 p<0.001 | 0.798 p<0.001 | 0.876 p<0.001 | 0.865 p<0.001 | 0.902 p<0.001 | 0.950 p<0.001 | 0.947 p<0.001 | 0.963 p<0.001 | 0.981 p<0.001 | 0.986 p<0.001 | 1 |

Table S7. Correlation matrix for brachycephalic BOAS grade 1 group “G1”. Minimum temperature of thermal windows and physical parameters.

| Brachycephalic  BOAS Grade 1  (Minimum) | Distance | Time | Age | Weight | T°  Caruncle | T°  Upper eyelid | | T°  Lower eyelid | | T°  Upper lip | | T°  Lower lip | T°  Mas | | | T°  nostrl | | T°  Flimbs | | T°  Hlimbs | | T°  *Gracilis* | | T°  *Femoralis* | | T°  *Gastocnemio* | |  |
| --- | --- | --- | --- | --- | --- | --- | --- | --- | --- | --- | --- | --- | --- | --- | --- | --- | --- | --- | --- | --- | --- | --- | --- | --- | --- | --- | --- | --- |
| Distance | 1 |  |  |  |  | |  | |  | |  | | |  |  | |  | |  | |  | |  | |  | |  | |
| Time | 0.564 p<0.001 | 1 |  |  |  | |  | |  | |  | | |  |  | |  | |  | |  | |  | |  | |  | |
| Age | 0.213 p=0.186 | 0.217 p=0.178 | 1 |  |  | |  | |  | |  | | |  |  | |  | |  | |  | |  | |  | |  | |
| Weight | 0.204 p=0.207 | 0.228 p=0.157 | 0.890 p<0.001 | 1 |  | |  | |  | |  | | |  |  | |  | |  | |  | |  | |  | |  | |
| T°Caruncle | 0.254 p=0.119 | 0.728 p<0.001 | 0.322 p=0.045 | 0.274 p=0.092 | 1 | |  | |  | |  | | |  |  | |  | |  | |  | |  | |  | |  | |
| T°upper eyelid | 0.249 p=0.121 | 0.811 p<0.001 | 0.263 p=0.101 | 0.279 p=0.081 | 0.971 p<0.001 | | 1 | |  | |  | | |  |  | |  | |  | |  | |  | |  | |  | |
| T°Lower eyelid | 0.205 p=0.205 | 0.768 p<0.001 | 0.353 p=0.026 | 0.344 p=0.030 | 0.988 p<0.001 | | 0.984 p<0.001 | | 1 | |  | | |  |  | |  | |  | |  | |  | |  | |  | |
| T°Upper lip | 0.128 p=0.430 | 0.780 p<0.001 | 0.322 p=0.043 | 0.320 p=0.044 | 0.956 p<0.001 | | 0.972 p<0.001 | | 0.978 p<0.001 | | 1 | | |  |  | |  | |  | |  | |  | |  | |  | |
| T°Lower lip | 0.144 p=0.376 | 0.752 p<0.001 | 0.272 p=0.089 | 0.292 p=0.068 | 0.969 p<0.001 | | 0.979 p<0.001 | | 0.980 p<0.001 | | 0.976 p<0.001 | | | 1 |  | |  | |  | |  | |  | |  | |  | |
| T°Mas | 0.201 p=0.215 | 0.766 p<0.001 | 0.309 p=0.052 | 0.302 p=0.058 | 0.980 p<0.001 | | 0.984 p<0.001 | | 0.987 p<0.001 | | 0.980 p<0.001 | | | 0.984 p<0.001 | 1 | |  | |  | |  | |  | |  | |  | |
| T°nostril | 0.111 p=0.497 | 0.740 p<0.001 | 0.306 p=0.055 | 0.332 p=0.036 | 0.963 p<0.001 | | 0.968 p<0.001 | | 0.966 p<0.001 | | 0.982 p<0.001 | | | 0.982 p<0.001 | 0.971 p<0.001 | | 1 | |  | |  | |  | |  | |  | |
| T°Flimbs | 0.228 p=0.157 | 0.775 p<0.001 | 0.314 p=0.049 | 0.325 p=0.041 | 0.969 p<0.001 | | 0.981 p<0.001 | | 0.981 p<0.001 | | 0.978 p<0.001 | | | 0.986 p<0.001 | 0.987 p<0.001 | | 0.979 p<0.001 | | 1 | |  | |  | |  | |  | |
| T°Hlimbs | 0.181 p=0.264 | 0.767 p<0.001 | 0.284 p=0.076 | 0.277 p=0.084 | 0.941 p<0.001 | | 0.970 p<0.001 | | 0.954 p<0.001 | | 0.964 p<0.001 | | | 0.966 p<0.001 | 0.979 p<0.001 | | 0.966 p<0.001 | | 0.978 p<0.001 | | 1 | |  | |  | |  | |
| T°*Gracilis* | 0.070 p=0.667 | 0.761 p<0.001 | 0.246 p=0.125 | 0.263 p=0.100 | 0.919 p<0.001 | | 0.959 p<0.001 | | 0.951 p<0.001 | | 0.981 p<0.001 | | | 0.972 p<0.001 | 0.965 p<0.001 | | 0.978 p<0.001 | | 0.966 p<0.001 | | 0.971 p<0.001 | | 1 | |  | |  | |
| T°*Femoralis* | 0.022 p=0.892 | 0.717 p<0.001 | 0.332 p=0.036 | 0.329 p=0.038 | 0.937 p<0.001 | | 0.950 p<0.001 | | 0.959 p<0.001 | | 0.982 p<0.001 | | | 0.969 p<0.001 | 0.962 p<0.001 | | 0.979 p<0.001 | | 0.963 p<0.001 | | 0.965 p<0.001 | | 0.980 p<0.001 | | 1 | |  | |
| T°*Gastocnemio* | 0.113 p=0.487 | 0.812 p<0.001 | 0.230 p=0.154 | 0.242 p=0.132 | 0.885 p<0.001 | | 0.946 p<0.001 | | 0.934 p<0.001 | | 0.973 p<0.001 | | | 0.944 p<0.001 | 0.943 p<0.001 | | 0.954 p<0.001 | | 0.946 p<0.001 | | 0.951 p<0.001 | | 0.987 p<0.001 | | 0.962 p<0.001 | | 1 | |

Table S8. Correlation matrix for brachycephalic BOAS grade 1 group “G1”. Maximum temperature of thermal windows and physical parameters.

| Brachycephalic  BOAS Grade 1  (Maximum) | Distance | Time | Age | Weight | T°  Caruncle | T°  Upper eyelid | T°  Lower eyelid | T°  Upper lip | T°  Lower lip | T°  Mas | T°  nostril | | T°  Flimbs | T°  Hlimbs | T°  *Gracilis* | | T°  *Femoralis* | T°  *Gastocnemio* |
| --- | --- | --- | --- | --- | --- | --- | --- | --- | --- | --- | --- | --- | --- | --- | --- | --- | --- | --- |
| Distance | 1 |  |  |  |  |  |  |  |  |  |  |  | |  | |  |  |  |
| Time | 0.564 p<0.001 | 1 |  |  |  |  |  |  |  |  |  |  | |  | |  |  |  |
| Age | 0.213 p=0.186 | 0.217 p=0.178 | 1 |  |  |  |  |  |  |  |  |  | |  | |  |  |  |
| Weight | 0.204 p=0.207 | 0.228 p=0.157 | 0.890 p<0.001 | 1 |  |  |  |  |  |  |  |  | |  | |  |  |  |
| T°Caruncle | 0.093 p=0.570 | 0.766 p<0.001 | 0.306 p=0.055 | 0.292 p=0.068 | 1 |  |  |  |  |  |  |  | |  | |  |  |  |
| T°Upper eyelid | 0.170 p=0.293 | 0.732 p<0.001 | 0.319 p=0.045 | 0.316 p=0.047 | 0.974 p<0.001 | 1 |  |  |  |  |  |  | |  | |  |  |  |
| T°Lower eyelid | 0.114 p=0.482 | 0.677 p<0.001 | 0.361 p=0.022 | 0.341 p=0.031 | 0.952 p<0.001 | 0.974 p<0.001 | 1 |  |  |  |  |  | |  | |  |  |  |
| T°Upper lip | 0.076 p=0.640 | 0.730 p<0.001 | 0.314 p=0.048 | 0.297 p=0.063 | 0.988 p<0.001 | 0.986 p<0.001 | 0.964 p<0.001 | 1 |  |  |  |  | |  | |  |  |  |
| T°Lower lip | 0.075 p=0.644 | 0.718 p<0.001 | 0.303 p=0.057 | 0.317 p=0.046 | 0.980 p<0.001 | 0.974 p<0.001 | 0.939 p<0.001 | 0.976 p<0.001 | 1 |  |  |  | |  | |  |  |  |
| T°Mas | 0.295 p=0.064 | 0.762 p<0.001 | 0.322 p=0.042 | 0.317 p=0.046 | 0.930 p<0.001 | 0.978 p<0.001 | 0.938 p<0.001 | 0.950 p<0.001 | 0.932 p<0.001 | 1 |  |  | |  | |  |  |  |
| T°nostril | 0.230 p=0.154 | 0.771 p<0.001 | 0.287 p=0.073 | 0.289 p=0.070 | 0.949 p<0.001 | 0.978 p<0.001 | 0.936 p<0.001 | 0.961 p<0.001 | 0.950 p<0.001 | 0.986 p<0.001 | 1 |  | |  | |  |  |  |
| T°Flimbs | 0.120 p=0.462 | 0.725 p<0.001 | 0.336 p=0.034 | 0.346 p=0.029 | 0.968 p<0.001 | 0.983 p<0.001 | 0.945 p<0.001 | 0.978 p<0.001 | 0.982 p<0.001 | 0.960 p<0.001 | 0.967 p<0.001 | 1 | |  | |  |  |  |
| T°Hlimbs | 0.101 p=0.536 | 0.738 p<0.001 | 0.326 p=0.040 | 0.341 p=0.031 | 0.988 p<0.001 | 0.981 p<0.001 | 0.957 p<0.001 | 0.983 p<0.001 | 0.990 p<0.001 | 0.941 p<0.001 | 0.954 p<0.001 | 0.985 p<0.001 | | 1 | |  |  |  |
| T°*Gracilis* | 0.158 p=0.330 | 0.774 p<0.001 | 0.293 p=0.067 | 0.297 p=0.063 | 0.984 p<0.001 | 0.984 p<0.001 | 0.946 p<0.001 | 0.985 p<0.001 | 0.972 p<0.001 | 0.964 p<0.001 | 0.973 p<0.001 | 0.972 p<0.001 | | 0.981 p<0.001 | | 1 |  |  |
| T°*Femoralis* | 0.044 p=0.789 | 0.699 p<0.001 | 0.332 p=0.036 | 0.334 p=0.035 | 0.983 p<0.001 | 0.981 p<0.001 | 0.966 p<0.001 | 0.992 p<0.001 | 0.975 p<0.001 | 0.941 p<0.001 | 0.944 p<0.001 | 0.978 p<0.001 | | 0.985 p<0.001 | | 0.978 p<0.001 | 1 |  |
| T°*Gastocnemio* | 0.223 p=0.167 | 0.832 p<0.001 | 0.252 p=0.116 | 0.250 p=0.120 | 0.965 p<0.001 | 0.938 p<0.001 | 0.879 p<0.001 | 0.946 p<0.001 | 0.948 p<0.001 | 0.918 p<0.001 | 0.933 p<0.001 | 0.930 p<0.001 | | 0.952 p<0.001 | | 0.969 p<0.001 | 0.936 p<0.001 | 1 |

Table S9. Correlation matrix for brachycephalic BOAS grade 1 group “G1”. Average temperature of thermal windows and physical parameters.

| Brachycephalic  BOAS Grade 1  (AVERAGE) | Distance | Time | Age | Weight | T°  Caruncle | T°  Upper eyelid | T°  Lower eyelid | T°  Upper lip | T°  Lower lip | T°  Mas | T°  nostril | T°  Flimbs | T°  Hlimbs | T°  *Gracilis* | T°  *Femoralis* | T°  *Gastocnemio* |
| --- | --- | --- | --- | --- | --- | --- | --- | --- | --- | --- | --- | --- | --- | --- | --- | --- |
| Distance | 1 |  |  |  |  |  |  |  |  |  |  |  |  |  |  |  |
| Time | 0.564 p<0.001 | 1 |  |  |  |  |  |  |  |  |  |  |  |  |  |  |
| Age | 0.213 p=0.186 | 0.217 p=0.178 | 1 |  |  |  |  |  |  |  |  |  |  |  |  |  |
| Weight | 0.204 p=0.207 | 0.228 p=0.157 | 0.890 p<0.001 | 1 |  |  |  |  |  |  |  |  |  |  |  |  |
| T°Caruncle | 0.173 p=0.286 | 0.728 p<0.001 | 0.331 p=0.037 | 0.320 p=0.044 | 1 |  |  |  |  |  |  |  |  |  |  |  |
| T°Upper eyelid | 0.244 p=0.129 | 0.738 p<0.001 | 0.364 p=0.021 | 0.358 p=0.023 | 0.971 p<0.001 | 1 |  |  |  |  |  |  |  |  |  |  |
| T°Lower eyelid | 0.139 p=0.391 | 0.680 p<0.001 | 0.376 p=0.017 | 0.358 p=0.023 | 0.985 p<0.001 | 0.954 p<0.001 | 1 |  |  |  |  |  |  |  |  |  |
| T°Upper lip | 0.039 p=0.810 | 0.721 p<0.001 | 0.313 p=0.049 | 0.312 p=0.050 | 0.969 p<0.001 | 0.949 p<0.001 | 0.952 p<0.001 | 1 |  |  |  |  |  |  |  |  |
| T°Lower lip | 0.141 p=0.387 | 0.766 p<0.001 | 0.314 p=0.048 | 0.326 p=0.040 | 0.956 p<0.001 | 0.958 p<0.001 | 0.918 p<0.001 | 0.977 p<0.001 | 1 |  |  |  |  |  |  |  |
| T°Mas | 0.204 p=0.208 | 0.744 p<0.001 | 0.363 p=0.021 | 0.353 p=0.025 | 0.978 p<0.001 | 0.984 p<0.001 | 0.974 p<0.001 | 0.964 p<0.001 | 0.952 p<0.001 | 1 |  |  |  |  |  |  |
| T°nostril | 0.198 p=0.222 | 0.775 p<0.001 | 0.291 p=0.069 | 0.316 p=0.047 | 0.963 p<0.001 | 0.966 p<0.001 | 0.919 p<0.001 | 0.965 p<0.001 | 0.990 p<0.001 | 0.954 p<0.001 | 1 |  |  |  |  |  |
| T°Flimbs | 0.16 p=0.324 | 0.718 p<0.001 | 0.352 p=0.026 | 0.348 p=0.028 | 0.969 p<0.001 | 0.978 p<0.001 | 0.945 p<0.001 | 0.968 p<0.001 | 0.982 p<0.001 | 0.968 p<0.001 | 0.981 p<0.001 | 1 |  |  |  |  |
| T°Hlimbs | 0.113 p=0.487 | 0.759 p<0.001 | 0.290 p=0.070 | 0.297 p=0.062 | 0.967 p<0.001 | 0.959 p<0.001 | 0.932 p<0.001 | 0.986 p<0.001 | 0.992 p<0.001 | 0.963 p<0.001 | 0.987 p<0.001 | 0.979 p<0.001 | 1 |  |  |  |
| T°*Gracilis* | 0.161 p=0.322 | 0.785 p<0.001 | 0.308 p=0.053 | 0.314 p=0.049 | 0.968 p<0.001 | 0.966 p<0.001 | 0.939 p<0.001 | 0.984 p<0.001 | 0.990 p<0.001 | 0.967 p<0.001 | 0.983 p<0.001 | 0.980 p<0.001 | 0.992 p<0.001 | 1 |  |  |
| T°*Femoralis* | 0.03 p=0.856 | 0.686 p<0.001 | 0.363 p=0.021 | 0.342 p=0.031 | 0.960 p<0.001 | 0.945 p<0.001 | 0.943 p<0.001 | 0.985 p<0.001 | 0.974 p<0.001 | 0.957 p<0.001 | 0.955 p<0.001 | 0.978 p<0.001 | 0.981 p<0.001 | 0.975 p<0.001 | 1 |  |
| T°*Gastocnemio* | 0.19 p=0.239 | 0.817 p<0.001 | 0.283 p=0.077 | 0.277 p=0.083 | 0.928 p<0.001 | 0.928 p<0.001 | 0.881 p<0.001 | 0.957 p<0.001 | 0.977 p<0.001 | 0.926 p<0.001 | 0.969 p<0.001 | 0.946 p<0.001 | 0.980 p<0.001 | 0.977 p<0.001 | p<0.001 | 1 |

Table S10. Correlation matrix for brachycephalic BOAS grade 2 group “G2”. Minimum temperature of thermal windows and physical parameters.

| Brahycephalic BOAS Grade 2  (Minimum) | Distance | Time | Age | Weight | T°  Caruncle | T°  Upper eyelid | T°  Lower eyelid | T°  Upper lip | T°  Lower lip | T°  Mas | T°  nostril | T°  Flimbs | T°  Hlimbs | T°  *Gracilis* | T°  *Femoralis* | T°  *Gastocnemio* |
| --- | --- | --- | --- | --- | --- | --- | --- | --- | --- | --- | --- | --- | --- | --- | --- | --- |
| Distance | 1 |  |  |  |  |  |  |  |  |  |  |  |  |  |  |  |
| Time | 0.492 p<0.001 | 1 |  |  |  |  |  |  |  |  |  |  |  |  |  |  |
| Age | 0.132 p=0.269 | 0.182 p=0.127 | 1 |  |  |  |  |  |  |  |  |  |  |  |  |  |
| Weight | 0.143 p=0.229 | 0.184 p=0.121 | 0.962 p<0.001 | 1 |  |  |  |  |  |  |  |  |  |  |  |  |
| T°Caruncle | 0.217 p=0.068 | 0.843 p<0.001 | 0.294 p=0.012 | 0.297 p=0.011 | 1 |  |  |  |  |  |  |  |  |  |  |  |
| T°upper eyelid | 0.167 p=0.161 | 0.836 p<0.001 | 0.289 p=0.014 | 0.292 p=0.013 | 0.990 p<0.001 | 1 |  |  |  |  |  |  |  |  |  |  |
| T°Lower eyelid | 0.181 p=0.127 | 0.828 p<0.001 | 0.324 p=0.006 | 0.326 p=0.005 | 0.994 p<0.001 | 0.990 p<0.001 | 1 |  |  |  |  |  |  |  |  |  |
| T°Upper lip | 0.103 p=0.391 | 0.757 p<0.001 | 0.334 p=0.004 | 0.349 p=0.003 | 0.971 p<0.001 | 0.967 p<0.001 | 0.980 p<0.001 | 1 |  |  |  |  |  |  |  |  |
| T°Lower lip | 0.104 p=0.384 | 0.813 p<0.001 | 0.309 p=0.008 | 0.311 p=0.008 | 0.987 p<0.001 | 0.990 p<0.001 | 0.988 p<0.001 | 0.969 p<0.001 | 1 |  |  |  |  |  |  |  |
| T°Mas | 0.015 p=0.900 | 0.782 p<0.001 | 0.322 p=0.006 | 0.319 p=0.006 | 0.966 p<0.001 | 0.976 p<0.001 | 0.968 p<0.001 | 0.955 p<0.001 | 0.989 p<0.001 | 1 |  |  |  |  |  |  |
| T°nostril | 0.224 p=0.059 | 0.817 p<0.001 | 0.316 p=0.007 | 0.319 p=0.006 | 0.983 p<0.001 | 0.976 p<0.001 | 0.984 p<0.001 | 0.971 p<0.001 | 0.967 p<0.001 | 0.936 p<0.001 | 1 |  |  |  |  |  |
| T°Flimbs | 0.222 p=0.061 | 0.847 p<0.001 | 0.307 p=0.009 | 0.305 p=0.009 | 0.993 p<0.001 | 0.990 p<0.001 | 0.992 p<0.001 | 0.965 p<0.001 | 0.982 p<0.001 | 0.961 p<0.001 | 0.987 p<0.001 | 1 |  |  |  |  |
| T°Hlimbs | 0.218 p=0.066 | 0.824 p<0.001 | 0.305 p=0.009 | 0.310 p=0.008 | 0.981 p<0.001 | 0.977 p<0.001 | 0.984 p<0.001 | 0.969 p<0.001 | 0.976 p<0.001 | 0.95 p<0.001 | 0.977 p<0.001 | 0.981 p<0.001 | 1 |  |  |  |
| T°*Gracilis* | 0.228 p=0.054 | 0.853 p<0.001 | 0.313 p=0.007 | 0.321 p=0.006 | 0.983 p<0.001 | 0.978 p<0.001 | 0.985 p<0.001 | 0.974 p<0.001 | 0.969 p<0.001 | 0.943 p<0.001 | 0.988 p<0.001 | 0.986 p<0.001 | 0.974 p<0.001 | 1 |  |  |
| T°*Femoralis* | 0.067 p=0.576 | 0.814 p<0.001 | 0.270 p=0.022 | 0.271 p=0.021 | 0.976 p<0.001 | 0.984 p<0.001 | 0.977 p<0.001 | 0.963 p<0.001 | 0.991 p<0.001 | 0.990 p<0.001 | 0.948 p<0.001 | 0.969 p<0.001 | 0.968 p<0.001 | 0.956 p<0.001 | 1 |  |
| T°*Gastocnemio* | 0.061 p=0.609 | 0.809 p<0.001 | 0.278 p=0.018 | 0.280 p=0.017 | 0.974 p<0.001 | 0.983 p<0.001 | 0.976 p<0.001 | 0.963 p<0.001 | 0.990 p<0.001 | 0.987 p<0.001 | 0.950 p<0.001 | 0.969 p<0.001 | 0.967 p<0.001 | 0.958 p<0.001 | 0.997 p<0.001 | 1 |

Table S11. Correlation matrix for brachycephalic BOAS grade 2 group “G2”. Maximum temperature of thermal windows and physical parameters.

| Brachycephalic  BOAS grade 2  (Maximum) | Distance | Time | Age | Weight | T°  Caruncle | T°  Upper eyelid | T°  Lower eyelid | T°  Upper lip | T°  Lower lip | T°  Mas | T°  nostril | T°  Flimbs | T°  Hlimbs | T°  *Gracilis* | T°  *Femoralis* | T°  *Gastocnemio* |
| --- | --- | --- | --- | --- | --- | --- | --- | --- | --- | --- | --- | --- | --- | --- | --- | --- |
| Distance | 1 |  |  |  |  |  |  |  |  |  |  |  |  |  |  |  |
| Time | 0.492 p<0.001 | 1 |  |  |  |  |  |  |  |  |  |  |  |  |  |  |
| Age | 0.132 p=0.269 | 0.182 p=0.127 | 1 |  |  |  |  |  |  |  |  |  |  |  |  |  |
| Weight | 0.143 p=0.229 | 0.184 p=0.121 | 0.962 p<0.001 | 1 |  |  |  |  |  |  |  |  |  |  |  |  |
| T°Caruncle | 0.115 p=0.335 | 0.789 p<0.001 | 0.318 p=0.006 | 0.330 p=0.005 | 1 |  |  |  |  |  |  |  |  |  |  |  |
| T°Upper eyelid | 0.156 p=0.191 | 0.828 p<0.001 | 0.324 p=0.006 | 0.325 p=0.005 | 0.990 p<0.001 | 1 |  |  |  |  |  |  |  |  |  |  |
| T°Lower eyelid | 0.150 p=0.208 | 0.759 p<0.001 | 0.323 p=0.006 | 0.337 p=0.004 | 0.989 p<0.001 | 0.979 p<0.001 | 1 |  |  |  |  |  |  |  |  |  |
| T°Upper lip | 0.131 p=0.274 | 0.755 p<0.001 | 0.321 p=0.006 | 0.334 p=0.004 | 0.990 p<0.001 | 0.984 p<0.001 | 0.992 p<0.001 | 1 |  |  |  |  |  |  |  |  |
| T°Lower lip | 0.110 p=0.356 | 0.797 p<0.001 | 0.305 p=0.009 | 0.313 p=0.007 | 0.990 p<0.001 | 0.990 p<0.001 | 0.972 p<0.001 | 0.985 p<0.001 | 1 |  |  |  |  |  |  |  |
| T°Mas | 0.103 p=0.389 | 0.813 p<0.001 | 0.320 p=0.006 | 0.311 p=0.008 | 0.963 p<0.001 | 0.975 p<0.001 | 0.933 p<0.001 | 0.953 p<0.001 | 0.979 p<0.001 | 1 |  |  |  |  |  |  |
| T°nostril | 0.322 p=0.006 | 0.780 p<0.001 | 0.323 p=0.006 | 0.335 p=0.004 | 0.947 p<0.001 | 0.947 p<0.001 | 0.971 p<0.001 | 0.959 p<0.001 | 0.924 p<0.001 | 0.890 p<0.001 | 1 |  |  |  |  |  |
| T°Flimbs | 0.194 p=0.102 | 0.795 p<0.001 | 0.340 p=0.003 | 0.343 p=0.003 | 0.972 p<0.001 | 0.977 p<0.001 | 0.967 p<0.001 | 0.972 p<0.001 | 0.971 p<0.001 | 0.965 p<0.001 | 0.963 p<0.001 | 1 |  |  |  |  |
| T°Hlimbs | 0.166 p=0.164 | 0.788 p<0.001 | 0.339 p=0.004 | 0.343 p=0.003 | 0.987 p<0.001 | 0.988 p<0.001 | 0.984 p<0.001 | 0.989 p<0.001 | 0.984 p<0.001 | 0.957 p<0.001 | 0.965 p<0.001 | 0.985 p<0.001 | 1 |  |  |  |
| T°*Gracilis* | 0.242 p=0.040 | 0.787 p<0.001 | 0.322 p=0.006 | 0.339 p=0.004 | 0.977 p<0.001 | 0.975 p<0.001 | 0.990 p<0.001 | 0.984 p<0.001 | 0.963 p<0.001 | 0.920 p<0.001 | 0.980 p<0.001 | 0.963 p<0.001 | 0.980 p<0.001 | 1 |  |  |
| T°*Femoralis* | ´-0.102 p=0.395 | 0.136 p=0.256 | 0.267 p=0.023 | 0.275 p=0.019 | 0.254 p=0.032 | 0.280 p=0.017 | 0.246 p=0.037 | 0.285 p=0.015 | 0.312 p=0.008 | 0.340 p=0.003 | 0.226 p=0.057 | 0.336 p=0.004 | 0.288 p=0.014 | 0.270 p=0.022 | 1 |  |
| T°*Gastocnemio* | 0.119 p=0.320 | 0.776 p<0.001 | 0.307 p=0.009 | 0.320 p=0.006 | 0.990 p<0.001 | 0.984 p<0.001 | 0.985 p<0.001 | 0.989 p<0.001 | 0.981 p<0.001 | 0.952 p<0.001 | 0.958 p<0.001 | 0.979 p<0.001 | 0.993 p<0.001 | 0.975 p<0.001 | 0.261 p=0.027 | 1 |

Table S12. Correlation matrix for brachycephalic BOAS grade 2 group “G2”. Average temperature of thermal windows and physical parameters.

| Brachycephalic  BOAS Grade 2  (AVERAGE) | Distance | Time | Age | Weight | T°  Caruncle | T°  Upper eyelid | T°  Lower eyelid | T°  Upper lip | T°  Lower lip | T°  Mas | T°  nostril | T°  Flimbs | T°  Hlimbs | T°  *Gracilis* | T°  *Femoralis* | T°  *Gastocnemio* |
| --- | --- | --- | --- | --- | --- | --- | --- | --- | --- | --- | --- | --- | --- | --- | --- | --- |
| Distance | 1 |  |  |  |  |  |  |  |  |  |  |  |  |  |  |  |
| Time | 0.492 p<0.001 | 1 |  |  |  |  |  |  |  |  |  |  |  |  |  |  |
| Age | 0.132 p=0.269 | 0.182 p=0.127 | 1 |  |  |  |  |  |  |  |  |  |  |  |  |  |
| Weight | 0.143 p=0.229 | 0.184 p=0.121 | 0.962 p<0.001 | 1 |  |  |  |  |  |  |  |  |  |  |  |  |
| T°Caruncle | 0.155 p=0.194 | 0.783 p<0.001 | 0.332 p=0.004 | 0.341 p=0.003 | 1 |  |  |  |  |  |  |  |  |  |  |  |
| T°Upper eyelid | 0.161 p=0.176 | 0.839 p<0.001 | 0.310 p=0.008 | 0.309 p=0.008 | 0.983 p<0.001 | 1 |  |  |  |  |  |  |  |  |  |  |
| T°Lower eyelid | 0.182 p=0.125 | 0.803 p<0.001 | 0.336 p=0.004 | 0.343 p=0.003 | 0.994 p<0.001 | 0.985 p<0.001 | 1 |  |  |  |  |  |  |  |  |  |
| T°Upper lip | 0.108 p=0.366 | 0.753 p<0.001 | 0.347 p=0.003 | 0.361 p=0.002 | 0.991 p<0.001 | 0.975 p<0.001 | 0.987 p<0.001 | 1 |  |  |  |  |  |  |  |  |
| T°Lower lip | 0.112 p=0.350 | 0.775 p<0.001 | 0.329 p=0.005 | 0.345 p=0.003 | 0.992 p<0.001 | 0.984 p<0.001 | 0.988 p<0.001 | 0.989 p<0.001 | 1 |  |  |  |  |  |  |  |
| T°Mas | ´-0.002 p=0.389 | 0.759 p<0.001 | 0.317 p=0.007 | 0.320 p=0.006 | 0.954 p<0.001 | 0.958 p<0.001 | 0.953 p<0.001 | 0.951 p<0.001 | 0.960 p<0.001 | 1 |  |  |  |  |  |  |
| T°nostril | 0.272 p=0.021 | 0.800 p<0.001 | 0.322 p=0.006 | 0.330 p=0.005 | 0.971 p<0.001 | 0.953 p<0.001 | 0.969 p<0.001 | 0.954 p<0.001 | 0.957 p<0.001 | 0.899 p<0.001 | 1 |  |  |  |  |  |
| T°Flimbs | 0.207 p=0.081 | 0.815 p<0.001 | 0.343 p=0.003 | 0.339 p=0.004 | 0.981 p<0.001 | 0.977 p<0.001 | 0.981 p<0.001 | 0.972 p<0.001 | 0.970 p<0.001 | 0.951 p<0.001 | 0.976 p<0.001 | 1 |  |  |  |  |
| T°Hlimbs | 0.173 p=0.147 | 0.787 p<0.001 | 0.335 p=0.004 | 0.339 p=0.004 | 0.992 p<0.001 | 0.982 p<0.001 | 0.987 p<0.001 | 0.990 p<0.001 | 0.985 p<0.001 | 0.941 p<0.001 | 0.967 p<0.001 | 0.981 p<0.001 | 1 |  |  |  |
| T°*Gracilis* | 0.239 p=0.043 | 0.825 p<0.001 | 0.315 p=0.007 | 0.330 p=0.005 | 0.983 p<0.001 | 0.978 p<0.001 | 0.986 p<0.001 | 0.972 p<0.001 | 0.975 p<0.001 | 0.911 p<0.001 | 0.981 p<0.001 | 0.970 p<0.001 | 0.982 p<0.001 | 1 |  |  |
| T°*Femoralis* | 0.048 p=0.686 | 0.819 p<0.001 | 0.267 p=0.023 | 0.269 p=0.022 | 0.964 p<0.001 | 0.984 p<0.001 | 0.965 p<0.001 | 0.955 p<0.001 | 0.968 p<0.001 | 0.971 p<0.001 | 0.919 p<0.001 | 0.950 p<0.001 | 0.959 p<0.001 | 0.953 p<0.001 | 1 |  |
| T°*Gastocnemio* | 0.112 p=0.350 | 0.796 p<0.001 | 0.309 p=0.008 | 0.321 p=0.006 | 0.990 p<0.001 | 0.985 p<0.001 | 0.987 p<0.001 | 0.981 p<0.001 | 0.986 p<0.001 | 0.968 p<0.001 | 0.952 p<0.001 | 0.969 p<0.001 | 0.982 p<0.001 | 0.975 p<0.001 | 0.985 p<0.001 | 1 |

Table S13. Correlation matrix for brachycephalic BOAS grade 3 group “G3”. Minimum temperature of thermal windows and physical parameters.

| Brachycephalic  BOAS Grade 3  (Minimum) | Distance | Time | Age | Weight | T°  Caruncle | T°  Upper eyelid | T°  Lower eyelid | T°  Upper lip | T°  Lower lip | T°  Mas | | T°  nostril | | T°  Flimbs | | T°  Hlimbs | | T°  *Gracilis* | | T°  *Femoralis* | | T°  *Gastocnemio* | |  |
| --- | --- | --- | --- | --- | --- | --- | --- | --- | --- | --- | --- | --- | --- | --- | --- | --- | --- | --- | --- | --- | --- | --- | --- | --- |
| Distance | 1 |  |  |  |  |  |  |  |  | |  | |  | |  | |  | |  | |  | |  | |
| Time | 0.555 p<0.001 | 1 |  |  |  |  |  |  |  | |  | |  | |  | |  | |  | |  | |  | |
| Age | 0.242 p=0.097 | 0.174 p=0.238 | 1 |  |  |  |  |  |  | |  | |  | |  | |  | |  | |  | |  | |
| Weight | 0.239 p=0.101 | 0.173 p=0.240 | 0.871 p<0.001 | 1 |  |  |  |  |  | |  | |  | |  | |  | |  | |  | |  | |
| T°Caruncle | 0.344 p=0.017 | 0.820 p<0.001 | 0.277 p=0.057 | 0.265 p=0.069 | 1 |  |  |  |  | |  | |  | |  | |  | |  | |  | |  | |
| T°Upper eyelid | 0.275 p=0.059 | 0.876 p<0.001 | 0.242 p=0.098 | 0.241 p=0.100 | 0.961 p<0.001 | 1 |  |  |  | |  | |  | |  | |  | |  | |  | |  | |
| T°Lower eyelid | 0.265 p=0.069 | 0.817 p=<.001 | 0.292 p=0.044 | 0.282 p=0.052 | 0.972 p<0.001 | 0.969 p<0.001 | 1 |  |  | |  | |  | |  | |  | |  | |  | |  | |
| T°Upper lip | 0.203 p=0.166 | 0.820 p<0.001 | 0.311 p=0.032 | 0.289 p=0.046 | 0.969 p<0.001 | 0.984 p<0.001 | 0.981 p<0.001 | 1 |  | |  | |  | |  | |  | |  | |  | |  | |
| T°Lower lip | 0.299 p=0.039 | 0.850 p<0.001 | 0.282 p=0.052 | 0.276 p=0.058 | 0.976 p<0.001 | 0.989 p<0.001 | 0.984 p<0.001 | 0.986 p<0.001 | 1 | |  | |  | |  | |  | |  | |  | |  | |
| T°Mas | 0.222 p=0.129 | 0.861 p<0.001 | 0.243 p=0.096 | 0.232 p=0.113 | 0.916 p<0.001 | 0.980 p<0.001 | 0.927 p<0.001 | 0.965 p<0.001 | 0.965 p<0.001 | | 1 | |  | |  | |  | |  | |  | |  | |
| T°nostril | 0.175 p=0.233 | 0.819 p<0.001 | 0.297 p=0.040 | 0.277 p=0.057 | 0.945 p<0.001 | 0.984 p<0.001 | 0.968 p<0.001 | 0.987 p<0.001 | 0.972 p<0.001 | | 0.963 p<0.001 | | 1 | |  | |  | |  | |  | |  | |
| T°Flimbs | 0.169 p=0.251 | 0.840 p<0.001 | 0.260 p=0.074 | 0.259 p=0.075 | 0.896 p<0.001 | 0.972 p<0.001 | 0.914 p<0.001 | 0.958 p=<.001 | 0.947 p<0.001 | | 0.986 p<0.001 | | 0.966 p<0.001 | | 1 | |  | |  | |  | |  | |
| T°Hlimbs | 0.099 p=0.504 | 0.823 p<0.001 | 0.215 p=0.143 | 0.211 p=0.149 | 0.853 p<0.001 | 0.956 p<0.001 | 0.889 p<0.001 | 0.938 p<0.001 | 0.926 p<0.001 | | 0.980 p<0.001 | | 0.950 p<0.001 | | 0.984 p<0.001 | | 1 | |  | |  | |  | |
| T°*Gracilis* | 0.158 p=0.285 | 0.867 p<0.001 | 0.218 p=0.136 | 0.218 p=0.136 | 0.867 p<0.001 | 0.963 p<0.001 | 0.897 p<0.001 | 0.942 p<0.001 | 0.932 p<0.001 | | 0.981 p<0.001 | | 0.953 p<0.001 | | 0.986 p<0.001 | | 0.991 p<0.001 | | 1 | |  | |  | |
| T°*Femoralis* | 0.116 p=0.433 | 0.832 p<0.001 | 0.273 p=0.061 | 0.261 p=0.073 | 0.891 p<0.001 | 0.971 p<0.001 | 0.929 p<0.001 | 0.965 p<0.001 | 0.950 p<0.001 | | 0.980 p<0.001 | | 0.974 p<0.001 | | 0.988 p<0.001 | | 0.988 p<0.001 | | 0.986 p<0.001 | | 1 | |  | |
| T°*Gastocnemio* | ´-0.002 p=0.991 | 0.792 p<0.001 | 0.217 p=0.139 | 0.206 p=0.160 | 0.805 p<0.001 | 0.918 p<0.001 | 0.855 p<0.001 | 0.909 p<0.001 | 0.882 p<0.001 | | 0.945 p<0.001 | | 0.925 p<0.001 | | 0.953 p<0.001 | | 0.983 p<0.001 | | 0.974 p<0.001 | | 0.974 p<0.001 | | 1 | |

Table S14. Correlation matrix for brachycephalic BOAS grade 3 group “G3”. Maximum temperature of thermal windows and physical parameters.

| Brachycephalic  BOAS Grade 3  (Maximum) | Distance | Time | Age | Weight | T°  Caruncle | T°  Upper eyelid | T°  Lower eyelid | T°  Upper lip | T°  Lower lip | T°  Mas | T°  nostril | T°  Flimbs | T°  Hlimbs | T°  *Gracilis* | T°  *Femoralis* | T°  *Gastocnemio* |
| --- | --- | --- | --- | --- | --- | --- | --- | --- | --- | --- | --- | --- | --- | --- | --- | --- |
| Distance | 1 |  |  |  |  |  |  |  |  |  |  |  |  |  |  |  |
| Time | 0.555 p<0.001 | 1 |  |  |  |  |  |  |  |  |  |  |  |  |  |  |
| Age | 0.242 p=0.097 | 0.174 p=0.238 | 1 |  |  |  |  |  |  |  |  |  |  |  |  |  |
| Weight | 0.239 p=0.101 | 0.173 p=0.240 | 0.871 p<0.001 | 1 |  |  |  |  |  |  |  |  |  |  |  |  |
| T°Caruncle | 0.230 p=0.116 | 0.847 p<0.001 | 0.314 p=0.030 | 0.296 p=0.041 | 1 |  |  |  |  |  |  |  |  |  |  |  |
| T°Upper eyelid | 0.165 p=0.261 | 0.833 p<0.001 | 0.281 p=0.053 | 0.263 p=0.071 | 0.988 p<0.001 | 1 |  |  |  |  |  |  |  |  |  |  |
| T°Lower eyelid | 0.237 p=0.104 | 0.843 p<0.001 | 0.280 p=0.054 | 0.274 p=0.059 | 0.983 p<0.001 | 0.980 p<0.001 | 1 |  |  |  |  |  |  |  |  |  |
| T°Upper lip | 0.239 p=0.102 | 0.766 p<0.001 | 0.318 p=0.028 | 0.301 p=0.037 | 0.936 p<0.001 | 0.924 p<0.001 | 0.973 p<0.001 | 1 |  |  |  |  |  |  |  |  |
| T°Lower lip | 0.276 p=0.057 | 0.760 p<0.001 | 0.348 p=0.015 | 0.313 p=0.030 | 0.944 p<0.001 | 0.936 p<0.001 | 0.971 p<0.001 | 0.980 p<0.001 | 1 |  |  |  |  |  |  |  |
| T°Mas | 0.354 p=0.013 | 0.880 p<0.001 | 0.199 p=0.175 | 0.195 p=0.183 | 0.958 p<0.001 | 0.959 p<0.001 | 0.970 p<0.001 | 0.922 p<0.001 | 0.943 p<0.001 | 1 |  |  |  |  |  |  |
| T°nostril | 0.250 p=0.086 | 0.657 p<0.001 | 0.304 p=0.036 | 0.277 p=0.057 | 0.844 p<0.001 | 0.832 p<0.001 | 0.892 p<0.001 | 0.945 p<0.001 | 0.944 p<0.001 | 0.849 p<0.001 | 1 |  |  |  |  |  |
| T°Flimbs | 0.023 p=0.877 | 0.762 p<0.001 | 0.293 p=0.043 | 0.272 p=0.062 | 0.965 p<0.001 | 0.978 p<0.001 | 0.948 p<0.001 | 0.889 p<0.001 | 0.896 p<0.001 | 0.901 p<0.001 | 0.781 p<0.001 | 1 |  |  |  |  |
| T°Hlimbs | 0.034 p=0.820 | 0.800 p<0.001 | 0.228 p=0.119 | 0.218 p=0.137 | 0.956 p<0.001 | 0.970 p<0.001 | 0.933 p<0.001 | 0.862 p<0.001 | 0.850 p<0.001 | 0.895 p<0.001 | 0.729 p<0.001 | 0.985 p<0.001 | 1 |  |  |  |
| T°*Gracilis* | 0.129 p=0.382 | 0.851 p<0.001 | 0.271 p=0.062 | 0.242 p=0.098 | 0.964 p<0.001 | 0.969 p<0.001 | 0.942 p<0.001 | 0.876 p<0.001 | 0.859 p<0.001 | 0.901 p<0.001 | 0.753 p<0.001 | 0.965 p<0.001 | 0.983 p<0.001 | 1 |  |  |
| T°*Femoralis* | 0.022 p=0.882 | 0.775 p<0.001 | 0.281 p=0.053 | 0.290 p=0.046 | 0.953 p<0.001 | 0.957 p<0.001 | 0.920 p<0.001 | 0.850 p<0.001 | 0.842 p<0.001 | 0.878 p<0.001 | 0.724 p<0.001 | 0.977 p<0.001 | 0.983 p<0.001 | 0.971 p<0.001 | 1 |  |
| T°*Gastocnemio* | 0.122 p=0.409 | 0.851 p<0.001 | 0.229 p=0.118 | 0.211 p=0.150 | 0.971 p<0.001 | 0.977 p<0.001 | 0.952 p<0.001 | 0.886 p<0.001 | 0.874 p<0.001 | 0.916 p<0.001 | 0.760 p<0.001 | 0.974 p<0.001 | 0.989 p<0.001 | 0.991 p<0.001 | 0.970 p<0.001 | 1 |

Table S15. Correlation matrix for brachycephalic BOAS grade 3 group “G3”. Average temperature of thermal windows and physical parameters.

| Brachycephalic  BOAS Grade 3  (AVERAGE) | Distance | Time | Age | Weight | T°  Caruncle | T°  Upper eyelid | T°  Lower eyelid | T°  Upper lip | T°  Lower lip | T°  Mas | T°  nostril | T°  Flimbs | | T°  Hlimbs | | T°  *Gracilis* | | T°  *Femoralis* | | T°  *Gastocnemio* | |
| --- | --- | --- | --- | --- | --- | --- | --- | --- | --- | --- | --- | --- | --- | --- | --- | --- | --- | --- | --- | --- | --- |
| Distance | 1 |  |  |  |  |  |  |  |  |  |  | |  | |  |  |  | |  | |  |
| Time | 0.555 p<0.001 | 1 |  |  |  |  |  |  |  |  |  | |  | |  |  |  | |  | |  |
| Age | 0.242 p=0.097 | 0.174 p=0.238 | 1 |  |  |  |  |  |  |  |  | |  | |  |  |  | |  | |  |
| Weight | 0.239 p=0.101 | 0.173 p=0.240 | 0.871 p<0.001 | 1 |  |  |  |  |  |  |  | |  | |  |  |  | |  | |  |
| T°Caruncle | 0.278 p=0.056 | 0.847 p<0.001 | 0.288 p=0.047 | 0.270 p=0.063 | 1 |  |  |  |  |  |  | |  | |  |  |  | |  | |  |
| T°Upper eyelid | 0.196 p=0.183 | 0.862 p<0.001 | 0.259 p=0.076 | 0.249 p=0.087 | 0.963 p<0.001 | 1 |  |  |  |  |  | |  | |  |  |  | |  | |  |
| T°Lower eyelid | 0.222 p=0.130 | 0.835 p<0.001 | 0.273 p=0.060 | 0.262 p=0.072 | 0.992 p<0.001 | 0.971 p<0.001 | 1 |  |  |  |  | |  | |  |  |  | |  | |  |
| T°Upper lip | 0.199 p=0.175 | 0.829 p<0.001 | 0.299 p=0.039 | 0.283 p=0.052 | 0.985 p<0.001 | 0.980 p<0.001 | 0.992 p<0.001 | 1 |  |  |  | |  | |  |  |  | |  | |  |
| T°Lower lip | 0.284 p=0.050 | 0.811 p<0.001 | 0.312 p=0.031 | 0.287 p=0.048 | 0.98 p<0.001 | 0.944 p<0.001 | 0.982 p<0.001 | 0.978 p<0.001 | 1 |  |  | |  | |  |  |  | |  | |  |
| T°Mas | 0.331 p=0.022 | 0.878 p<0.001 | 0.224 p=0.125 | 0.204 p=0.165 | 0.966 p<0.001 | 0.973 p<0.001 | 0.964 p<0.001 | 0.961 p<0.001 | 0.954 p<0.001 | 1 |  | |  | |  |  |  | |  | |  |
| T°nostril | 0.247 p=0.091 | 0.758 p<0.001 | 0.290 p=0.046 | 0.270 p=0.064 | 0.975 p<0.001 | 0.903 p<0.001 | 0.970 p<0.001 | 0.954 p<0.001 | 0.973 p<0.001 | 0.922 p<0.001 | 1 | |  | |  |  |  | |  | |  |
| T°Flimbs | 0.094 p=0.525 | 0.800 p<0.001 | 0.259 p=0.075 | 0.253 p=0.082 | 0.938 p<0.001 | 0.982 p<0.001 | 0.950 p<0.001 | 0.970 p<0.001 | 0.917 p<0.001 | 0.940 p<0.001 | 0.882 p<0.001 | | 1 | |  |  |  | |  | |  |
| T°Hlimbs | 0.097 p=0.511 | 0.842 p<0.001 | 0.211 p=0.150 | 0.212 p=0.147 | 0.930 p<0.001 | 0.982 p<0.001 | 0.950 p<0.001 | 0.957 p<0.001 | 0.899 p<0.001 | 0.933 p<0.001 | 0.864 p<0.001 | | 0.977 p<0.001 | | 1 |  |  | |  | |  |
| T°*Gracilis* | 0.152 p=0.302 | 0.867 p<0.001 | 0.247 p=0.090 | 0.237 p=0.106 | 0.923 p<0.001 | 0.976 p<0.001 | 0.941 p<0.001 | 0.947 p<0.001 | 0.886 p<0.001 | 0.928 p<0.001 | 0.854 p<0.001 | | 0.962 p<0.001 | | 0.991 p<0.001 | 1 |  | |  | |  |
| T°*Femoralis* | 0.044 p=0.766 | 0.788 p<0.001 | 0.284 p=0.050 | 0.277 p=0.056 | 0.932 p<0.001 | 0.978 p<0.001 | 0.951 p<0.001} | 0.964 p<0.001 | 0.910 p<0.001 | 0.925 p<0.001 | 0.880 p<0.001 | | 0.986 p<0.001 | | 0.985 p<0.001 | 0.974 p<0.001 | 1 | |  | |  |
| T°*Gastocnemio* | 0.065 p=0.660 | 0.831 p<0.001 | 0.239 p=0.101 | 0.222 p=0.129 | 0.903 p<0.001 | 0.966 p<0.001 | 0.926 p<0.001 | 0.933 p<0.001 | 0.866 p<0.001 | 0.900 p<0.001 | 0.832 p<0.001 | | 0.956 p<0.001 | | 0.990 p<0.001 | 0.989 p<0.001 | 0.973 p<0.001 | | 1 | |  |
